# Plasma-activated water as a wound rinse solution in patients with diabetes-related foot ulcers in two Australian hospitals: study protocol for a phase I double-blinded, randomised controlled trial

**DOI:** 10.1136/bmjopen-2026-118420

**Published:** 2026-07-10

**Authors:** Adrian I Abdo, Neil Mcmillan, Li Lao, Guilherme N Pena, Robert Fitridge, Katharina Richter

**Affiliations:** 1 School of Pharmacy and Biomedical Science, College of Health, Adelaide University, Adelaide, South Australia, Australia; 2 Basil Hetzel Institute for Translational Health Research, The Queen Elizabeth Hospital, Adelaide University, Adelaide, South Australia, Australia; 3 Study Group for Biofilms (ESGB), European Society of Clinical Microbiology and Infectious Diseases, Basel, Switzerland; 4 School of Medicine, College of Health, Adelaide University, Adelaide, South Australia, Australia; 5 Department of Vascular and Endovascular Surgery, Royal Adelaide Hospital, Adelaide, South Australia, Australia; 6 Department of Vascular and Endovascular surgery, Flinders Medical Centre, Bedford Park, South Australia, Australia; 7 Institute for Photonics and Advanced Sensing, Adelaide University, Adelaide, South Australia, Australia

**Keywords:** WOUND MANAGEMENT, Diabetic foot, Clinical Trial, Clinical Protocols, Anti-Bacterial Agents

## Abstract

**Introduction:**

Patients living with diabetes-related foot ulcers (DFU) often have non-healing wounds that, despite ongoing clinical management and wound debridement as standard of care, can proceed to infection and lower limb amputation. Additionally, treatment guidelines do not recommend most topical antibiotics or antiseptic treatments, limiting complements to surgical wound debridement. We present a protocol for a phase I randomised controlled trial testing the safety and efficacy of plasma-activated water as an antiseptic for preventing infection and promoting ulcer healing.

**Methods and analysis:**

This protocol describes a single-centre, two-armed (1:1 allocation), double-blinded, randomised controlled trial of plasma-activated water (PAW) for the treatment of non-healing DFU. 20 participants will be randomised to the control (standard care with 10-minute treatment with saline-soaked gauze twice weekly for 6 weeks) or intervention group (same but with 10-minute treatment with PAW-soaked gauze instead). Primary outcome is the safety and tolerability of PAW in treating non-healing wounds, while secondary outcome is the effect on promoting wound healing and antimicrobial activity.

**Ethics and dissemination:**

The Central Adelaide Local Health Network Human Research Ethics Committee (2025/HRE00207) has approved the study. Results will be presented at international conferences and published in a peer-reviewed clinical journal.

**Trial registration number:**

This trial was registered in the Australia New Zealand Clinical Trials Registry (registration number ACTRN12625000902493) on 20 August 2025.

**STRENGTHS AND LIMITATIONS OF THIS STUDY**

- This is a double-blinded, randomised controlled trial design that strongly curbs potential for bias.
- Study participants will come from a population already receiving ongoing care by the investigating clinical team, improving likelihood of study compliance throughout the study period.
- As the primary outcome is safety, no sample size analysis was performed, so the secondary outcome analysis is likely to be underpowered.
- There is potential that plasma-activated water (PAW) could cause pain and feel different to saline from participant’s experience, which would unblind the participant and clinical investigator.

## Introduction

### Diabetes-related foot disease

 Diabetes-related foot disease is defined by the International Working Group on the Diabetic Foot (IWGDF) guidelines as “Disease of the foot of a person with current or previously diagnosed diabetes mellitus that includes one or more of the following: peripheral neuropathy, peripheral artery disease, infection, ulcer(s), neuro‐osteoarthropathy, gangrene, or amputation.”^1^ Diabetes-related foot ulcers (DFU) are usually accompanied by peripheral neuropathy and/or peripheral artery disease in the lower extremity and are a common and costly condition requiring hospitalisation and frequent ongoing clinical management. In people with diabetes, just the presence of a foot ulcer has been shown to increase risk of mortality by nearly 50% compared with diabetes without a foot ulcer.^2^

Despite the tertiary level multidisciplinary team (MDT) efforts in diabetic foot disease treatment and ulcer prevention, around 25% of patients with diabetes have a lifetime risk of developing a foot wound, of which over 50% of those will develop infection, and over 25% of those infections will become chronic/relapsing infections.^3^ Infections in these patients carry a significantly higher risk of progressing to necrosis and osteomyelitis, requiring surgical debridement or amputation. These outcomes severely impair patient quality of life and increase morbidity and mortality. In addition to the major psychological trauma of a diabetes-related amputation, mortality is notoriously high with a 5-year mortality rate of 70% for all patients with diabetes and 2-year mortality of 74% for those undergoing dialysis.^3^,^6^

### Challenges to treatment and treatment gap

Even with rigid compliance to frequent wound treatment protocols enacted by tertiary level MDT, incidence of infections and the subsequent risks of amputation are still high in patients with diabetes-related foot disease. The current 2023 IWGDF guidelines do not recommend prophylactic use of topical antibiotics or antiseptics (eg, silver, honey, bacteriophages at the site of diabetes-related foot infection (DFI; recommendation 11, 23 and 24)).^1 7 8^ These recommendations stem from the low quality of evidence, high risk of bias and evidence that most topical treatments either do not benefit or impede wound healing.^8^ This leaves limited non-surgical strategies available for clinicians to complement the surgical debridement of wounds. Consequently, one of the IWGDF guidelines stated a key area of concern is the potential for topical antimicrobial administration to limit the use of systemic antibiotics,^8^ for which there is low evidence of reducing risk of developing infection and improving wound healing, and could result in potentially harmful and avoidable adverse effects of the treatment.^9^ Plasma-activated water (PAW) could potentially benefit patients with DFU by directly promoting wound healing and helping prevent infection. This could also help limit reliance on systemic antibiotic use and reduce the associated risks of adverse effects, costs and propagation of antimicrobial resistance.

### Plasma-activated water

PAW is produced by discharging cold plasma into water, generating a complex chemical solution containing several short-lived reactive oxygen and nitrogen species (RONS), including hydroxyl (^•^OH), superoxide (O_2_^•−^) and nitric oxide (NO^•^) radicals, singlet oxygen (^1^O_2_), hydroxide (OH^−^) and peroxynitrite (ONOO^−^) ions.^10^,^12^ These quickly react in solution to form long-lived RONS such as nitrate/nitric acid (NO_3_^−^/HNO_3_), nitrite/nitrous acid (NO_2_^−^/HNO_2_) and hydrogen peroxide (H_2_O_2_). This results in an acidic pH, high oxidation-reduction potential and conductivity that creates an antimicrobial environment to kill microbes,^13^,^16^ while also eliciting cell signalling responses that promote wound healing in skin cells.

There are several murine wound model studies that support the clinical testing of PAW. Two independent studies showed that PAW significantly accelerated wound healing and had reduced inflammation in rats and mice with full thickness excisional wounds compared with untreated animals.^17 18^ PAW also led to faster wound healing in mice compared with medical alcohol-treated or vehicle-treated mice, while modulating interleukin (IL)−1β and IL-6 inflammatory cytokines and promoting vascular endothelial growth factor expression to promote neovascularisation in the wound.^19^ In a mouse burn wound infection model with methicillin-resistant *Staphylococcus aureus*, twice-daily topical application of PAW significantly reduced bacterial infection from day 4 onwards with histological analysis showing significantly greater re-epithelialisation compared with vehicle treatment.^20^ Overall, these animal studies indicate that PAW is antimicrobial and capable of promoting wound healing, as opposed to most current antiseptic solutions that impair wound healing and are not recommended for treating chronic wounds.^8 21^

It must be emphasised that ‘PAW’ is an umbrella term for solutions that have various chemical compositions and physical properties. This means that PAW derived from different devices, cold plasma generator configurations or parameters are not directly comparable between studies and must be experimentally verified for each individual use case. Different PAW formulations have been through various clinical trials as an antimicrobial in mucosal epithelial tissue (vaginal, oral and bronchopulmonary). Patients with bacterial vaginosis treated with a single 1-minute treatment of PAW (n=46) were reported to experience greater bacterial reduction than the betadine spray group (n=40; cf. 23% vs 13%, respectively).^22^ No adverse events (AEs) were reported for PAW, but its safety for mucosal epithelial tissue is supported for a single spray which cannot be extrapolated to longer term repeated treatment safety, and the reported antimicrobial activity analysis was unblinded and semiquantitative, leaving it prone to investigator bias. Two randomised placebo-controlled trials also tested PAW (compared with saline) against SARS-CoV-2; one trial was a gargle to shorten viral shedding,^23^ and the other testing safety and efficacy of nebulised PAW to treat COVID-19-associated pneumonia following antiviral failure.^24^ Thrice-daily gargle of PAW over 3 days significantly reduced cumulative incidence of delta and omicron infection, shorter viral shedding duration and greater symptom relief with no discomfort or adverse effects reported compared with saline.^23^ As a treatment for COVID-19-associated pneumonia, twice-daily nebulised PAW treatment over 4 days led to greater chest CT improvement in PAW versus saline group (cf. 81.8% vs 33.3%), significantly greater disappearance of cough and 3 days’ shorter hospital stay compared with saline.^24^

Currently, all clinical trials of PAW have been single-centre, phase I randomised controlled trials, and so far, PAW has never been used as a therapeutic agent for treatment of wounds. Here, we propose a study protocol for a phase I trial of PAW in patients with DFU. The Standard Protocol Items: Recommendations for Interventional Trials 2025 reporting checklist is provided with this study protocol.

### Study aims and outcomes

The primary outcome measures will be the safety and tolerability of PAW during and up to 6 weeks after initiation of treatment. The secondary outcomes will involve measuring the antimicrobial effectiveness and effects on wound healing during treatment and at 12 and 18 weeks after initiation of treatment.

#### Primary outcomes

1. The primary safety analysis will be based on the incidence and severity of adverse reactions during the treatment period, including but not limited to skin irritation or maceration.
2. Assessment of safety will also be performed based on data collected up to 6 weeks after initiation of treatment.

#### Secondary outcomes

1. Days to complete ulcer healing.
2. Presence and severity of infection.
3. Changes in wound quality based on ulcer assessment.
4. Quality of life assessment (Cardiff Wound Impact Schedule).

## Methodology

### Study design

**Figure 1:**
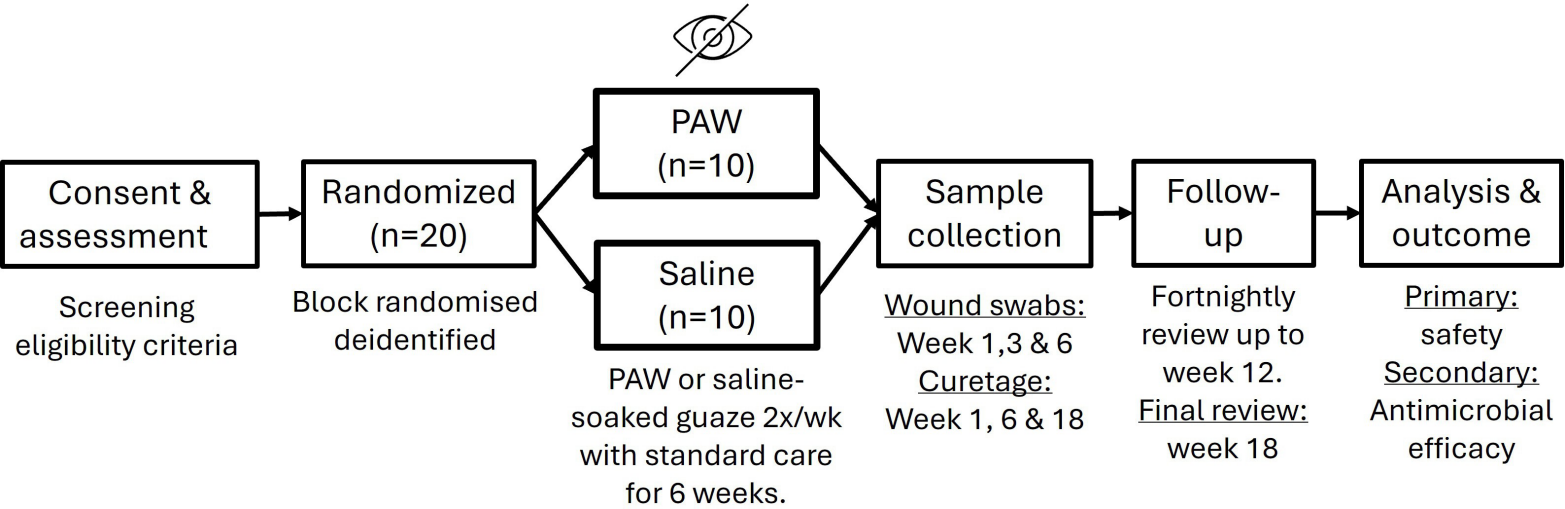
Clinical trial flow diagram. PAW, plasma-activated water.

This is a prospective, double-blinded, randomised, controlled parallel-treatment study to investigate the safety and tolerability of PAW in adults with DFU. Participants who provide consent to participate in the study and meet all inclusion criteria and no exclusion criteria will be randomly allocated to one of two treatment groups; standard wound care in addition to a temporary (10 min) gauze dressing soaked in either (1) PAW or (2) control (sterile saline). Saline will be used as a control as it is already used to clean wounds as a standard of care and is aqueous-based same as PAW treatment. Standard care will involve sharp debridement as clinically needed and determined at each visit, thorough wound bed preparation and applying dressings to absorb exudate and maintain a moist wound healing environment, and weight offloading devices to relieve foot pressure if needed, following the best practice for the type, severity and stage of ulcer individualised to each participant at the clinician’s discretion, regardless of group allocation. Standard care details will be recorded. Participants will attend a total of 12 scheduled visits over six consecutive weeks. A microbial swab will be taken by Levine technique at weeks 1 (start), 3 and 6 for measuring bacterial and fungal load. Curettages of the wound will be taken at weeks 1, 6 and 18, if available and if the wound is not yet healed. Follow-up wound review visits will then be conducted fortnightly until week 12 and a final review visit at week 18 (figure 1).

There will be no restrictions to the standard care participants would usually receive. The treatment groups will not replace antibiotic treatment as an intervention for any new infections that may arise. Participants will be informed that the experimental treatment will only be effective for the duration of the trial and that they will need to manage infections with their treating teams outside of the trial.

### Participant eligibility, recruitment and allocation

#### Recruitment

Participants will be recruited from the multidisciplinary foot (MDF) clinics and podiatry outpatient clinics at the Royal Adelaide Hospital and The Queen Elizabeth Hospital. Patients in these services are well known to clinical staff and the investigators. Potential participants will be identified by clinical staff and approached for participating in the trial by the patient’s attending clinician (vascular, nursing or podiatry).

Information will be provided by a clinical study investigator. Participants will be provided with a Participant Information Sheet and given up to 2 weeks to consider, have any questions answered or discuss with their family and friends before consenting. Participants may also be allowed to screen earlier if they choose and are deemed by the investigator to have adequately considered their options. Participants will be informed at every opportunity that participation or non-participation will not impact their care and that they are allowed to withdraw from the study at any time at their discretion.

#### Inclusion criteria

Prospective participants must be at least 18 years of age and are required to meet all of the following inclusion criteria to participate in the study:

1. Diagnosed and under current medical treatment for DM characterised by at least one of the following:
  1. HbA1c >6.5%.
  2. Fasting plasma glucose >7.0 mmol/L.
  3. Plasma glucose >11.1 mmol/L after 2 hour 75 g oral glucose tolerance test.
2. At least one cutaneous ulcer on the foot that has been under standard care for at least 4 weeks with an area between 2 and 20 cm^2^ at time of enrolment, equivalent to grades 1–2 on the Wound Infection foot Ischaemia (WIfI) wound clinical category.^25^ If a participant has ≥2 ulcers that meet the inclusion criteria, the ulcer with the largest area will be selected for the study and the other ulcers will receive standard care alone.
3. Ankle Brachial Index ≥0.4 and/or toe pressure >30 mmHg on the limb with study ulcer, equivalent to grade 2 or less on WIfI ischaemia category.^25^
4. Life expectancy of at least 6 months as assessed by the physician performing initial trial assessment.
5. The investigator believes the patient has the ability to follow the instruction on ulcer care.
6. Able to provide independent written informed consent, or witnessed by a family member or friend if cognitive impairment is suspected but the patient still satisfies all other criteria.

#### Exclusion criteria

The prospective participant will be excluded from the trial if they meet any of the following exclusion criteria:

1. Planned open or endovascular revascularisation, or any major or minor amputation of the index leg within the next 3 months.
2. Active moderate-to-severe infection in the study ulcer (WIfI foot infection category grade 2 or higher),^25^ or treatment with intravenous antibiotics within the past 2 weeks.
3. Clinical or radiographic sign of active osteomyelitis associated with the study ulcer.
4. Treatment with systemic immunosuppressants within 90 days of screening.
5. Active malignancy or history of malignancy within 5 years prior to screening (except for a past history of basal or squamous cell carcinomas).
6. History of HIV infection.
7. Participated in another trial within 60 days or within five half-lives of the last treatment (if the half-life of the investigational agent is known to be longer than 12 days) prior to the planned initiation of study treatment.
8. Any clinical condition or significant concurrent disease judged by the Investigator to complicate the evaluation of the trial treatment or contraindicate treatment.

**Figure 2:**
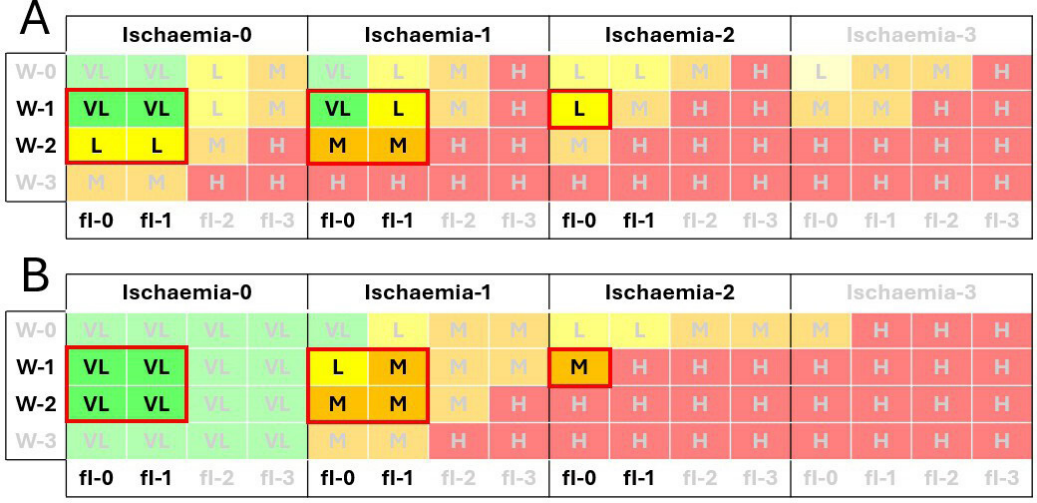
Wound Infection foot Ischaemia (WIfI) assessment risk matrix of (**A**) estimated risk of amputation at 1 year and (**B**) estimated likelihood of benefit of/requirement of revascularisation (adapted from WIfi^25^). Risk estimates are VL (very low), L (low), M (medium) and H (high) with risk stratification of prospective participants in this study highlighted.

Based on these criteria, grade scores for the WIfI wound ischaemia and foot infection categories place the prospective participants of the trial spanning the very low to medium risk category for estimated risk of amputation at 1 year and estimated likelihood of requiring revascularisation (figure 2).

### Patient and public involvement

We are grateful for advice from the Vascular Surgery Research Consumer Advisory panel for direction and feedback throughout the designing of our protocol. We are actively working with a Multidrug Resistant Organisms consumer advisor to facilitate liaising with consumers and providing lived experience for future trial design, including potentially as an investigator on future grants and clinical trials.

### Sample size

20 participants will be recruited in total. No power or sample size analysis has been performed, as this is a phase 1 study with safety as the primary outcome. Since there is no clinical information available for PAW as a topical treatment, and this is a simple two-armed trial, 10 participants per group is considered appropriate for a study determining safety as a qualitative primary outcome. Additional participants may be enrolled into the study at the discretion of the Principal Investigator to ensure at least 10 evaluable participants per treatment group. Participants who withdraw due to AEs will not be replaced.

### Allocation to treatment group

Participants will be randomly assigned to one of two treatment groups once written informed consent (patient information sheet and consent form V.1.1 attached as online supplemental file 1) has been obtained and eligibility established. Each participant will be allocated an identification (ID) number, so that participants can be identified without making assumptions about their subsequent eligibility for the study. Participants will be allocated to sequential, ascending 2-digit ID numbers (01, 02, 03…), which will provide a unique identifier for the duration of the study.

Treatments will be prepared according to a list of study IDs and assignments created from Microsoft Excel retained by investigator AIA, who at no time will have knowledge of patient identity or clinical information for any patient. Conversely, at no time will the other clinicians know the assignment of a patient to PAW or saline.

### Randomisation

Using Microsoft Excel, scientific investigator AIA will use a block randomisation procedure with three blocks of 10 participants with equal assignment to PAW and saline within each block. Allocation will be made by random number generation, randomisation will be locked prior to the trial starting, and only investigator AIA will have access to the randomisation sequence. Although only 20 participants are required, an additional block will be used in case of replacing participants who withdraw from the study.

### Blinding

All healthcare personnel, researchers and participants will be blinded to the assignment and treatment administered (double-blind) by using appropriately labelled de-identified treatments that will only be identifiable by participants’ study ID. Both treatments are a clear, colourless liquid that will be soaked in gauze before administration and so are unlikely to be distinguishable by care providers or participants. Only investigator AIA will prepare and retain knowledge of treatments based on preparation and linkage to participant study IDs on a password-protected Excel spreadsheet stored on a Central Adelaide Local Health Network (CALHN) device. This spreadsheet will be used to match for study safety and efficacy at the end of study, and/or once a suspected unexpected serious adverse reaction (SUSAR) has been confirmed.

### Observational data

#### Demographics and baseline clinical characteristics

Participant enrolment and disposition will be summarised for participants enrolled into the study and in each analysis set. The number of participants prematurely discontinuing from the study, along with the reason for early discontinuation, will also be summarised.

Demographic and baseline data recorded at screening will be summarised for all participants. Prior medications (taken on or before the first investigational product administration) and concomitant medications (taken after the first investigational product administration) will be recorded. Medications taken on the same day as investigational product administration will be considered both prior and concomitant medications.

#### Clinical laboratory measurements

Blood samples will be taken at screening and at 2 weeks, plus as clinically indicated from each participant. Laboratory results will be summarised for each treatment group using descriptive statistics at each scheduled visit for actual values and changes from baseline. Abnormal laboratory values will be flagged and will be identified in the listings. Microscopy data, if available, will be listed. The incidence of treatment-emergent abnormal laboratory findings will be summarised using frequency counts and percentages for each treatment group and listed separately.

1. Haematology: haemoglobin, haematocrit, red blood cell count, white blood cell count with differential, and platelet count.
2. Clinical chemistry: sodium, potassium, calcium, serum albumin, total protein, gamma glutamyl transferase, aspartate aminotransferase, alanine aminotransferase, alkaline phosphatase, total bilirubin, lactate dehydrogenase, glucose and creatinine. Creatinine clearance is calculated based on Cockcroft-Gault equation, not raw serum creatinine.
3. HbA1c will be performed at screening, unless recent result is available.

Wound swabs will be collected in Amies blue cap tubes and processed for colony-forming unit (CFU) count. LL will hand over samples to AIA or their delegate to process samples within 24 hours using standard microbiological techniques, and recording CFU/wound on a standard data collection sheet labelled by participant ID. Microbiological swabs will then be stored long term at −80 °C in saline with 25% glycerol:50% tryptone soya broth for biobanking. These biobank samples will be used for bacterial and fungal identification and isolation using selective and differential chromogenic agars. Bacterial and fungal isolates will be biobanked and tested for susceptibility to antimicrobials and PAW in vitro.

#### Vital signs analysis

Vital signs (including height, weight and body mass index) will be summarised for each treatment group using descriptive statistics at each protocol scheduled time point for actual values and change from baseline.

#### Wound characteristics

The clinical investigator will assess the characteristics of the study ulcer at each study visit based on standard-of-care measures of location, type (neuropathic, ischaemic, neuro-ischaemic), size (length, width, depth post debridement), involvement of deep structures, sinus tracts, tunnelling, wound bed tissue type, signs of infection, exudate (type and quantity), odour and pain.

#### Safety data and adverse events reporting

The study treatment information will be listed. The actual number of dressings applied will be summarised. AEs will be coded using the Medical Dictionary for Regulatory Activities (MedDRA). For each study treatment, number of participants, percentage of participants in the treatment group and numbers of TEAEs will be tabulated by system organ class and preferred term.

For the summaries of AEs, participants who experience the same AE (in terms of the MedDRA system organ class and preferred term) more than once will only be counted once for that event in the number of participants. Categorical summaries will include the frequency, incidences (one per participant) and percentages of events for participants and will be displayed by treatment group. Physical examination findings will be listed only.

#### Efficacy analyses

Efficacy indicators will be summarised descriptively with incidence rates or standard descriptive methods, as appropriate. Efficacy analysis will be based on data collected up to 12 and 18 weeks after initiation of treatment.

Assessment of efficacy will be based on the following outcome measures of the study ulcer: (1) days to complete ulcer healing and (2) changes in ulcer quality (eg, infection, signs, pain) on the Monash Diabetes Foot Assessment tool.

### Data analysis plan

For continuous variables, the mean±SD or 95% CI will be used to describe parametric data, while the median, minimum and maximum or IQR will be used to describe non-parametric data. Exploratory data analysis for the variables will be performed to ensure correct data input, visually and statistically analyse the normality of the data distribution (using histograms, quantile-quantile plots and D’Agostino-Pearson Omnibus test for the normal distribution) and find potential outliers, which will be excluded if values are greater than two SD from the mean for parametric data. For categorical variables, frequencies and percentages will be used to summarise data. Individual participant data will be presented in listings.

Efficacy indicators will be summarised descriptively with incidence rates or standard descriptive methods, as appropriate. Efficacy analysis will be based on data collected up to 18 weeks after initiation of treatment. One measure of efficacy will be the days to complete ulcer healing, to compare between groups by two-sample Welch’s t-test if data are parametric, or Wilcoxon-Mann-Whitney test if data are non-parametric, after normalising for baseline area to correct for ulcer size heterogeneity. Differences from start to end of the study in various ulcer qualities and quality of life assessments between each group will be analysed by Fisher’s exact test or χ^2^ test of independence. As a phase I study, the analysis of secondary efficacy indicators is likely to be underpowered due to low sample size. Therefore, the analyses of these results will not be used for statistical inference, but instead be used to perform power analysis and help determine effect sizes to inform sample size calculations for the presumed prospective phase II trial.

Once all data are collated and analysed, treatment groups will be unblinded (assuming no confirmed SUSAR previously unblinded the treatments). Investigators with competing interests are barred from any data analysis and interpretation contributing towards conference presentations and peer-review publications

### Study management plan, data storage and sample management

This study will use Research Electronic Data Capture (REDCap, Vanderbilt University, USA); a web-based program for secure data capture for research studies. Only approved CALHN researchers will be granted access to this database to add identifiable data, which will not be accessed by non-CALHN staff. Data will only be downloaded and shared with the University investigators and biostatistician in deidentified format; patient URN will be marked as an identifier in REDCap and removed from data exports. Procedure dates will be date-shifted in REDCap to maintain confidentiality. REDCap requires two-factor authentication to log in and a user is only able to view a project they created or a project they have been invited to.

All participant samples will be stored in reidentifiable format by study ID at the Basil Hetzel Institute for Translational Health Research, Adelaide, Australia. These samples will be owned by Adelaide University for staff and students to process and analyse as permitted by individual participant consent. Samples will be stored for up to 5 years to allow for other relevant studies to be performed. University investigator AIA or his delegate will be responsible for samples annotation and biobanking for storage periods, custodianship and management, and destruction of samples on schedule as biological waste.

Data are held by CALHN with NM as steward; blinding is performed by AIA without access to data. Analyses will be conducted in conjunction with independent Basil Hetzel Institute statistician under guidance from investigators, but excluding those with declared conflict of interest (including, but not limited to, KR). Safety unblinding will be allocated to a specific investigator who is not a principal investigator. Research monitoring where needed is provided by the institution (CALHN). A dedicated committee is not assigned as siloing already achieves its principal aims and the study itself is small.

### Dissemination

Participants will be notified of study results by the MDF clinic team as part of their ongoing care. Academic manuscripts will be published open access and in accordance with the Consolidated Standards of Reporting Trials 2025 guidelines for reporting randomised trials,^26^ with deidentified data made available. Authorship eligibility will be guided by the National Health and Medical Research Council (NHMRC) authorship guidelines in adherence to the Australian Code for the Responsible Conduct of Research (2018).

### Ethics and safety considerations

This study has received approval from the CALHN Human Research Ethics Committee (approval number 2025/HRE00207). All participants will be assigned a unique identification code that will be used for all data entry associated with this study to keep information strictly confidential. Only CALHN staff and not external investigators have access to participant identity or to any physical or electronic medical records. Only deidentified data will be shared with external investigators for analyses. Publications arising from this study will only contain analyses of deidentified, aggregated data.

#### Risks and benefits

As a first-in-human trial, some unforeseen risks to participants are unavoidable. As a novel agent applied to open wounds, the most likely adverse reaction is localised skin irritation. A care plan for these cases to limit impact of irritation on the wound microenvironment and healing will be enacted when these events occur. However, existing preclinical data highlights minimal risk to human cells and wound healing, and mouse models by our investigators showed no observed signs of pain or discomfort on application of PAW.^18^,^2027^ Furthermore, a large-animal (pig) preclinical study of full thickness incisional wounds treated twice weekly with PAW-soaked gauze for 3 weeks demonstrates no adverse effects (manuscript in preparation). As a temporary topical application, any systemic effects attributed to PAW are highly unlikely.

Our patients with DFU, who are predominantly neuropathic, have a decreased capacity to feel localised foot pain. If patients with incomplete loss of sensation do report any pain or discomfort from the rinse application, saline will immediately be applied to wash away PAW, the event reported as an adverse reaction and participant and investigator will naturally be unblinded to the treatment. Any instances of unblinding will be recorded, and when the participant reaches the end of the trial period, they will be asked to guess which group they were allocated to, in which they can answer (1) ‘control’, (2) ‘treatment’ or (3) ‘unsure’. The blinding index will then be calculated to determine blinding effectiveness.^28^ Given the marginal safety risks and the potential improvement to infection control using PAW that can also be effective against antibiotic-resistant organisms,^20 29 30^ we expect the potential benefits of the research for society to outweigh the risk of harm and discomfort.

We expect participants randomised to PAW treatment will have reduced bacterial load and recurrent infection during the study and preclinical data suggests that wound healing may be promoted for this group. Regardless, we expect participants in both groups to benefit from improved personalised care experienced by typical clinical trial participants.

## Discussion

Despite strong compliance with frequent wound treatment protocols enacted by tertiary level MDT of clinicians, the incidence of DFI and resulting risks of amputation are still high in patients with DFU. New adjuvant treatments to prevent infection to complement surgical debridement of wounds and promote wound healing are needed and are a key area of concern according to the IWGDF guidelines.^8^

This protocol outlines a safety trial that will determine the safety of PAW in treating DFU. If determined to be safe, the results of this trial will inform the development of future phase II clinical trial of the efficacy of PAW as a wound antiseptic solution for also promoting wound healing that can be readily implemented to current clinical management of DFU. As a first-in-use for the treatment of skin wounds in humans, this trial will also monitor participant’s experience to use of PAW (eg, presence of pain, quality of life changes). It is suspected that pain will be unlikely due to the high prevalence of peripheral neuropathy in these prospective participants.

### Strengths and limitations

This is a double-blinded, randomised controlled trial design that strongly curbs potential for bias. It is also highly likely that participants in the study will come from a population that are already receiving ongoing care by the investigating clinical team. This improves the chances that study participants will maintain compliance with treatments and follow-ups throughout the study period.

As this is a phase I study primarily for safety, no sample size analysis was performed, meaning any analyses of efficacy data is likely to be underpowered. This limits the interpretability of the efficacy of PAW for its antimicrobial activity and in promoting wound healing in this study. However, the efficacy results of this study will be used as pilot results to perform formal sample size analysis and to inform appropriate treatment groups for a prospective phase II study.

Although the study is a double-blinded design, there is the potential that PAW could cause pain and feel different to saline as a wound rinse from the participant’s experience. Inductive reasoning will then unblind the patient and clinical investigator, and the study will no longer be considered blinded.

### Trial status

This is the first version (V.1.01) of the study protocol (see online supplemental file 2), approved by the CALHN Human Research Ethics Committee (2025/HRE00207). Recruitment is expected to begin in February 2026 and the trial is expected to be completed by January 2027.

## Supplementary material

10.1136/bmjopen-2026-118420 online supplemental file 1

10.1136/bmjopen-2026-118420 online supplemental file 2

## References

[1] van Netten JJ, Bus SA, Apelqvist J (2024). Definitions and criteria for diabetes-related foot disease (IWGDF 2023 update). Diabetes Metab Res Rev.

[2] Anderson SG, Shoo H, Saluja S (2018). Social deprivation modifies the association between incident foot ulceration and mortality in type 1 and type 2 diabetes: a longitudinal study of a primary-care cohort. Diabetologia.

[3] Armstrong DG, Boulton AJM, Bus SA (2017). Diabetic Foot Ulcers and Their Recurrence. N Engl J Med.

[4] Lavery LA, Hunt NA, Ndip A (2010). Impact of Chronic Kidney Disease on Survival After Amputation in Individuals With Diabetes. Diabetes Care.

[5] Lazzarini PA, Hurn SE, Kuys SS (2017). The silent overall burden of foot disease in a representative hospitalised population. Int Wound J.

[6] Jeffcoate W, Boyko EJ, Game F (2024). Causes, prevention, and management of diabetes-related foot ulcers. Lancet Diabetes Endocrinol.

[7] Schaper NC, van Netten JJ, Apelqvist J (2024). Practical guidelines on the prevention and management of diabetes-related foot disease (IWGDF 2023 update). Diabetes Metab Res Rev.

[8] Senneville É, Albalawi Z, van Asten SA (2023). IWGDF/IDSA Guidelines on the Diagnosis and Treatment of Diabetes-related Foot Infections (IWGDF/IDSA 2023). Clin Infect Dis.

[9] Senneville É, Lipsky BA, Abbas ZG (2020). Diagnosis of infection in the foot in diabetes: a systematic review. Diabetes Metabolism Res.

[10] Kaushik NK, Ghimire B, Li Y (2018). Biological and medical applications of plasma-activated media, water and solutions. Biol Chem.

[11] Boehm D, Curtin J, Cullen PJ (2018). Hydrogen Peroxide and Beyond-the Potential of High-voltage Plasma-activated Liquids Against Cancerous Cells. Anticancer Agents Med Chem.

[12] Zhou R, Zhou R, Wang P (2020). Plasma-activated water: generation, origin of reactive species and biological applications. J Phys D: Appl Phys.

[13] Zhang Q, Liang Y, Feng H (2013). A study of oxidative stress induced by non-thermal plasma-activated water for bacterial damage. Appl Phys Lett.

[14] Tian Y, Ma R, Zhang Q (2015). Assessment of the Physicochemical Properties and Biological Effects of Water Activated by Non‐thermal Plasma Above and Beneath the Water Surface. Plasma Processes & Polymers.

[15] Kondeti V, Phan CQ, Wende K (2018). Long-lived and short-lived reactive species produced by a cold atmospheric pressure plasma jet for the inactivation of Pseudomonas aeruginosa and Staphylococcus aureus. Free Radic Biol Med.

[16] Ma M, Zhang Y, Lv Y (2020). The key reactive species in the bactericidal process of plasma activated water. J Phys D: Appl Phys.

[17] Lee HR, Kang SU, Kim HJ (2023). Liquid plasma as a treatment for cutaneous wound healing through regulation of redox metabolism. Cell Death Dis.

[18] Xu D, Wang S, Li B (2020). Effects of Plasma-Activated Water on Skin Wound Healing in Mice. Microorganisms.

[19] Wang S, Xu D, Qi M (2021). Plasma-Activated Water Promotes Wound Healing by Regulating Inflammatory Responses. *Biophysica*.

[20] Abdo AI, Nguyen HT, Kaul LD (2025). Plasma-activated water accelerates wound healing and reduces *Staphylococcus aureus* infection in vivo. *Biofilm*.

[21] Wilkins RG, Unverdorben M (2013). Wound cleaning and wound healing: a concise review. Adv Skin Wound Care.

[22] Jang Y, Bok J, Ahn DK (2022). Human Trial for the Effect of Plasma-Activated Water Spray on Vaginal Cleaning in Patients with Bacterial Vaginosis. Med Sci (Basel).

[23] Guo X, Lv X, Wu Y (2024). Safety and efficacy of plasma‐activated water on prolonged viral shedding of COVID‐19 patients: A randomized controlled trial. Plasma Processes & Polymers.

[24] Zhao H, Meng W, Lv X (2024). Nebulized inhalation of plasma-activated water in the treatment of progressive moderate COVID-19 patients with antiviral treatment failure: a randomized controlled pilot trial. BMC Infect Dis.

[25] Mills JL, Conte MS, Armstrong DG (2014). The Society for Vascular Surgery Lower Extremity Threatened Limb Classification System: Risk stratification based on Wound, Ischemia, and foot Infection (WIfI). J Vasc Surg.

[26] Hopewell S, Chan A-W, Collins GS (2025). CONSORT 2025 statement: Updated guideline for reporting randomised trials. PLoS Med.

[27] Abdo AI, Kopecki Z (2024). Comparing Redox and Intracellular Signalling Responses to Cold Plasma in Wound Healing and Cancer. Curr Issues Mol Biol.

[28] Bang H, Ni L, Davis CE (2004). Assessment of blinding in clinical trials. Control Clin Trials.

[29] Tan J, Karwe MV (2021). Inactivation and removal of Enterobacter aerogenes biofilm in a model piping system using plasma-activated water (PAW). *Innovative Food Science & Emerging Technologies*.

[30] Xu Z, Zhou X, Yang W (2020). In vitro antimicrobial effects and mechanism of air plasma‐activated water on *Staphylococcus aureus* biofilm. Plasma Processes & Polymers.

