# Supplementary material for: Plasma-activated water as a wound rinse solution in patients with diabetes-related foot ulcers in two Australian hospitals: study protocol for a phase I double-blinded, randomised controlled trial
Source: BMJ Open. 2026 Jul 10;16(7):e118420. doi: 10.1136/bmjopen-2026-118420 (PMC13358267; doi:10.1136/bmjopen-2026-118420)
Supplement: online supplemental file 1 [file bmjopen-16-7-s001.pdf]

Vascular Surgical Unit, Central Adelaide Local Health Network

HREC/CALHN/

Participant Information Sheet/Consent Form

**Title**	A Phase I randomised clinical trial of Plasma-Activated Water as a novel rinse agent for diabetes-related foot ulcers
**Principal Investigator**	Professor Robert Fitridge
**Associate Investigators**	A/Prof Katharina Richter<br>Dr Adrian Abdo<br>Dr Neil McMillan<br>Dr Guilherme Pena<br>Li Lao
**Location**	The Queen Elizabeth Hospital<br>Royal Adelaide Hospital

Part 1: What does my participation involve?

Introduction
You are invited to take part in the above-mentioned research project. This is because you
- are over 18 years of age
- have diabetes
- have at least one wound on your foot that has not healed.

The research project is testing a new treatment for treating diabetes-related foot wounds. This treatment is a 'wound rinse' called *plasma-activated water*.

This Participant Information Sheet/Consent Form tells you about the research project. It explains the tests and treatments involved. Knowing what is involved will help you decide if you want to take part in the research.

Please read this information carefully. Ask questions about anything that you don't understand or want to know more about. Before deciding whether or not to take part, you might want to talk about it with a relative, friend or your local doctor.

Participation in this research is voluntary. If you don't wish to take part, you don't have to. You will receive the best possible care whether or not you take part.

If you decide you want to take part in the research project, you will be asked to sign the consent section. By signing it you are telling us that you:

- Understand what you have read
- Consent to take part in the research project
- Consent to have the tests and treatments that are described
- Consent to the use of your personal and health information as described.

You will be given a copy of this Participant Information and Consent Form to keep.

Where is the research being done?

This study is being run within the high-risk foot services and vascular inpatient services at the Royal Adelaide Hospital and The Queen Elizabeth Hospital.

What is the purpose of this research?

Diabetes-related foot wounds often have many bacteria, which can cause clinical infection of the tissue and the bone underneath. These infections can be very difficult to treat, and can be why toes, feet, and legs need to be amputated. One of the reasons that these infections are very difficult to treat is because of antibiotic resistance, as well as the types of colonies that these bacteria create to protect themselves.

A/Prof Katharina Richter and Dr Adrian Abdo from the University of Adelaide have developed a new way to clean wounds in a way that does not promote antibiotic resistance and is effective against all types of bacteria. This treatment is called *plasma-activated water*, which involves running a plasma current like lightning through sterile water. After this activation, the water is shown to produce molecules that are toxic to all kinds of bacteria but not toxic to human cells. We are testing whether soaking wounds in this water will prevent infection.

This is a Phase 1 study. This activated water has been extensively studied in animals and cell cultures, but not in humans. This will be the first time that human patients will have their wounds treated with plasma-activated water. This is an experimental treatment, which means that it has not been approved for use by Regulatory Authorities such as Australia's Therapeutic Goods Administration (TGA). Experimental treatments may be tested in research studies such as this one.

What does participation in this research involve?

You will be participating in a randomised, controlled research project. Sometimes we do not know which treatment is best for treating a condition. To find out we need to compare different treatments. We put people into groups and give each group a different treatment. The results are compared to see if one is better. To try to make sure the groups are the same, each participant is put into a group by chance (random).
If you decide to join this study, you will be randomly assigned (by a computer) into one of the following two treatment groups:

- Group 1: Plasma-activated water will be poured on a dressing and applied to your wound prior to your other standard dressings.
- Group 2: Sterile saline (salt water) will be poured on a dressing and applied to your wound prior to your other standard dressings.

Because the water solution in both groups is colourless and odourless, you will not know what group you are in, and only certain members of the study team will know what group you are in.

If you agree to take part in this study, you will be asked to sign the Participant Consent Form. This study involves a minimum of one (1) data collection session at the start of this study. It may take up to two hours. All data collection sessions will happen in the high-risk foot services or inpatient services at Royal Adelaide Hospital or The Queen Elizabeth Hospital.

If you have more than one wound on your foot or feet, only one of them will be chosen as "the study wound". Your other wounds will continue to receive the dressings decided by your doctor or the wound care nurse.

Before you take part in this study, you will be given time to fully read and understand this information sheet and consent form. You will also be given the chance to ask any questions that you may have before you make the decision to participate. You can also discuss the study with your regular doctor, family and friends before you make a decision. You can take all the time you need to think about whether you wish to be involved.

If you decide to join the study, your participation will last for approximately 18 weeks: 2 weeks of observation, 4 weeks on study treatment, and 12 weeks of follow-up.

STUDY PROCEDURES
The following information describes what will happen to you during these study visits.

Screening Visit (Estimated duration: 1-2 hours)
You must meet certain criteria to be able to participate in this study. If you decide to be assessed for inclusion in the study, you will be asked to sign and date the consent form at the end of this document. You will then be asked some questions and have some tests to see if this study is suitable for you.

During the screening visit the following assessments and procedures will be conducted:

- Collection of your demographic information (which includes and any date of birth, age, gender, race and ethnicity).
- Review of your medical history (which includes your disease and surgical history, details about your wound(s) on your foot/feet or lower leg/legs as well as any medications you have been taking or still taking.
- A physical examination to assess your general appearance and health (including height and weight)
- Recording of your vital signs and blood pressure (including your blood pressure in both arms, heart rate, breathing rate and body temperature)
- Blood tests to assess your general health and glucose levels (approximately 5 mL of blood).
- Photographs and measurements of your wound (including size and appearance)

After the screening visit, the study doctor will determine your eligibility. If test results show that you have a new or worsening health problem, it may mean that you cannot take part in the study. If you agree and the study doctor thinks it is necessary, they may have to take extra samples to check the results.

If any health conditions that affect your ability to participate in this study are detected during screening, they will be discussed with you. If necessary, you will be referred for follow up visits with your general practitioner or medical specialist. If you are eligible, you will be asked to return to the study centre within two weeks of your screening visit.

Treatment and Evaluation Visits - Weeks 3 to 18 (Estimated duration: 1-1.5 hours)
You will receive wound rinse dressings on 8 occasions over 4 weeks. After the four weeks, you will return to standard care for the rest of the study.

There may be flexibility for some study procedures to be performed as a home visit by the wound trial nurse; please discuss this if you will have difficulty attending appointments.

At all scheduled visits your wound will be inspected, photographs of the wound will be taken, and your vital signs will be monitored. Blood and urine collections are also required to assess your general health. Details of these and other procedures conducted during the study are provided below.

Check of your signs, symptoms and medications
At all visits the study staff will ask if you have had any new symptoms or changes in your health or medications since your last visit. Your vital signs will be measured and, if required, a physical examination may be performed.

If any indications show that you have a new or worsening health problem, it may mean that you cannot continue to take part in the study. If you agree and the study doctor thinks it is necessary, they may have to take extra samples to check the results.

Wound pain
The study team will also ask you at each visit to assess the severity of any pain from the study wound. Pain will be rated from 0 (no pain) to 10 (worst possible pain).

Wound inspection and wound photographs
A member of the study team will check the study wound at each visit and record any changes since your last visit. Photographs of the wound will be taken at each visit and analysed to determine if there has been any change in the wound appearance.

Questionnaire
During the study (at Weeks 1, 6, and 18) you will be asked to complete a questionnaire about the impact your wound has had on your daily life. You will be asked how you are coping in both your daily life and social life, and about your wellbeing.

At each visit you will also be asked to provide an estimate of the time taken to care for your study wound, type and number of dressings used and how many clinic/home visits you have had since your last study visit. This information will assist in our understanding of the cost implications associated with treatment of diabetic foot/lower leg wounds.

Do I have to stop any of my regular medication to be involved?
During the study, you will continue to take your usual medications, or other medications that the study doctor recommends, to treat your foot wound or any other condition. You cannot participate in any other clinical trial for the duration of your involvement in this one.

Do I have to pay? Will I receive a Payment?
There are no additional costs associated with participating in this research project, nor will you be paid. All dressings, tests and medical care required as part of the research study will be provided to you free of charge.

Other relevant information about the research project
There will be approximately 20 participants enrolled in this study at the RAH and TQEH.

Do I have to take part in this research project?
Participation in any research project is voluntary. If you do not wish to take part, you do not have to. If you decide to take part and later change your mind, you are free to withdraw from the project at any stage.

Your decision whether to take part or not to take part, or to take part and then withdraw, will not affect your routine treatment, your relationship with those treating you or your relationship with the Royal Adelaide Hospital or The Queen Elizabeth Hospital. You will receive the best care whatever you decide.

What are the alternatives to participation?
You do not have to take part in this research study to receive treatment at this hospital. Other treatments are available including standard care options. Your study doctor will

discuss these with you before you decide whether to take part in this research study. You can also discuss the options with your local doctor.

What are the possible benefits of taking part?

We cannot guarantee or promise that you will receive any benefits from this research; however, the information we get from this research project may help us in the future to better treat people with foot and lower leg wounds.

What are the possible risks and disadvantages of taking part?

Medical treatments often cause side effects. In this study we may expect that you could experience mild swelling, redness, or irritation at the site where the treatment is applied. If you have any of these side effects, or are worried about them, talk with your study doctor. Your study doctor will also be looking out for side effects.

There may be side effects that the researchers do not expect or do not know about and that may be serious. Tell your study doctor immediately about any new or unusual symptoms that you get.

Many side effects go away shortly after treatment ends. However, sometimes side effects can be serious, long lasting or permanent. If a severe side effect or reaction occurs, your study doctor may need to stop your treatment. Your study doctor will discuss the best way of managing any side effects with you.

Can I withdraw from the study after agreeing to participate? What happens if I withdraw?

You may withdraw at any time and for any reason, using the withdrawal form provided at the end of this package or by speaking with a study investigator. If you decide to withdraw from the project, please notify a member of the research team before you withdraw. This notice will allow that person or the research supervisor to discuss any health risks or special requirements linked to withdrawing.
The investigator may ask what your reasons for withdrawal include – you do not have to provide this information, but it would help us to understand how best to improve this study if you are willing to share. You can request to have your data and samples to be destroyed as part of withdrawal; otherwise we will retain these up to the point that you withdraw. Withdrawing from the study will not have any impact on your ongoing treatment or relationship with your CALHN clinical team.

Part 2: How is the research project being conducted?

What will happen to information about me?

When you give Informed Consent, the Consent Forms, which will include your name, will be stored securely and separately from the data and only study staff will have access to these identified forms. The forms will be stored in a locked filing cabinet at the Royal Adelaide Hospital and The Queen Elizabeth Hospital, and will be retained for 15 years after conclusion of the study at the Basil Hetzel Institute for Translational Health Research.

The only information retained by the research group is history obtained at clinic visits, results of blood tests and any scans that may be ordered by your doctor, and wound examination results. These data will be collected using a random subject number and not your name, so you cannot be identified. All members of the study and collaborator team will have access to these de-identified data. A computer Registry database will also be set up on a password-protected server and only the study team will have access to these records. The electronic versions of your data will be stored for 15 years. At the end of this time, all paper records will be shredded securely and the computer database will be securely deleted.

It is anticipated that the results of this research project will be published and/or presented in a variety of forums. In any publication and/or presentation, information will be provided in such a way that you cannot be identified. This will be ensured by only reporting the amalgamated results of all participants, in addition to not recording your name in the database. All outcome results will be jointly owned by the collaborating institutions (CALHN, Western Sydney University, Central Coast Local Health District, The University of Adelaide).

In accordance with relevant Australian and/or South Australian privacy and other relevant laws, you have the right to request access to your information collected and stored by the research team. You also have the right to request that any information with which you disagree be corrected. Please contact a study team member named at the end of this document if you would like to access your information.

Complaints and compensation

Your participation in this study does not affect any rights you may have for compensation under common law. If you suffer any injuries or complications as a result of this research project, you should contact the study team as soon as possible and you will be assisted with arranging appropriate medical treatment. If you are eligible for Medicare, you can receive any medical treatment required to treat the injury or complication, free of charge, as a public patient in any Australian public hospital.

Who is organising and funding the research?

This research project is being conducted by Professor Robert Fitridge, Dr Guilherme Pena, and Dr Neil McMillan, in collaboration with clinicians and researchers at the University of Adelaide. No member of the research team will receive a personal financial benefit from your involvement in this research project (other than their ordinary wages). If knowledge acquired through this research leads to discoveries that are of commercial value to The Queen Elizabeth Hospital and Royal Adelaide Hospital, there will be no financial benefit to you or your family from these discoveries.

Conflicts of Interest: KR is a non-financial board member on the board of directors for RIBU Plasma Pty. Ltd. (Australia) and an inventor on patent WO/2024/013069 (Buske, C., & Richter, K. (2024). Process, apparatus and use of an apparatus for producing a plasma-activated liquid. Patent No. WO2024013069. World Intellectual Property Organization). KR will not analyse or interpret the data.

Who has reviewed the research project?

All research in Australia involving humans is reviewed by an independent group called a Human Research Ethics Committee (HREC). The ethical aspects of this research project have been approved by the HREC of CALHN. This project will be carried out according to the *National Statement on Ethical Conduct in Human Research (2007)*. This statement has been developed to protect the interests of people who agree to participate in human research studies.

Further information and who to contact

The person you may need to contact will depend on the nature of your query. If you want any further information about this project, you can contact either of the following people:

Principal Investigator

Name	Professor Robert Fitridge

Position	Professor of Vascular Surgery, CALHN
Telephone	8222 7711
Email	CALHNVascularDepartment@sa.gov.au

Study Coordinator

Name	Dr Neil McMillan
Position	Medical Scientist
Telephone	8222 7711
Email	neil.mcmillan@sa.gov.au

Reviewing HREC approving this research, HREC Executive Officer details and Complaints Contact

HREC Name	Central Adelaide Local Health Network Human Research Ethics Committee (CALHN HREC)
Contact	CALHN HREC Chair
Telephone	(08) 7117 2229
Email	Health.CALHNResearchEthics@sa.gov.au

Consent Form

**Title**	A Phase I randomised clinical trial of Plasma-Activated Water as a novel rinse agent for diabetes-related foot ulcers
**Principal Investigator**	Professor Robert Fitridge
**Associate Investigators**	A/Prof Katharina Richter<br>Dr Adrian Abdo<br>Dr Neil McMillan<br>Dr Guilherme Pena<br>Li Lao
**Location**	The Queen Elizabeth Hospital<br>Royal Adelaide Hospital

<u>Declaration by Participant</u>

I have read and understand that the study will be carried out as described in the Information Statement, a copy of which I have retained. I have been made aware of the procedures involved in the study, including any known or expected inconvenience, risk, discomfort, or potential side effect and of their implications as far as they are currently known by the researchers.
I understand that my participation in this study will allow the researchers and others, as described in the Information Statement, to have access to my medical record. I agree to this.
I agree to participate in the study and understand that I can withdraw at any time without providing a reason.
I understand that my personal information will remain confidential to the researchers.
I have had the opportunity to have questions answered to my satisfaction.
I hereby agree to participate in this research study.
I have read the Participant Information Sheet.
I understand the purposes, procedures and risks of the research described in the project.
I have had an opportunity to ask questions and I am satisfied with the answers I have received.
I freely agree to participate in this research project as described and understand that I am free to withdraw at any time during the study without affecting my future health care.
I understand that I will be given a signed copy of this document to keep.

I agree to my collected deidentified samples/data will be used in future research.
☐ Yes ☐ No - This specific research project
☐ Yes ☐ No - Other research that is closely related to this research project
☐ Yes ☐ No - Any future research.

Name of Participant (please print) ______________________________

Signature ______________________ Date ______________________

Declaration by Witness (if applicable)

I have been party to the verbal explanation of the research project, its procedures and risks and I believe that the participant has understood that explanation.

Name of Witness ______________________

Signature ______________________ Date ______________________

Relationship to Participant: ______________________

Declaration by Study Doctor/Nurse

I have given a verbal explanation of the research project, its procedures and risks and I believe that the participant has understood that explanation.

Name of Study Doctor /
Trial Nurse (please print) ______________________

Signature ______________________ Date ______________________

Government of South Australia
SA Health

THE UNIVERSITY of ADELAIDE

Vascular Surgical Unit, Central Adelaide Local Health Network

Form for Withdrawal of Participation

**Title**	A Phase I randomised clinical trial of Plasma-Activated Water as a novel rinse agent for diabetes-related foot ulcers
**Principal Investigator**	Professor Robert Fitridge
**Associate Investigators**	A/Prof Katharina Richter<br>Dr Adrian Abdo<br>Dr Neil McMillan<br>Dr Guilherme Pena<br>Li Lao
**Location**	The Queen Elizabeth Hospital<br>Royal Adelaide Hospital

Declaration by Participant

I wish to withdraw from participation in the above research project and understand that such withdrawal will not affect my routine treatment, my relationship with those treating me or my relationship with The Queen Elizabeth Hospital or Royal Adelaide Hospital.

Name of Participant (please print) ______________________________

Signature ______________________ Date ______________________

☐ Please destroy all data and samples related to my participation in this study.

Declaration by Study Doctor / Trial Nurse

I have given a verbal explanation of the implications of withdrawal from the research project and I believe that the participant has understood that explanation.

Name of Study Doctor /
Trial Nurse (please print) ______________________________

Signature ______________________ Date ______________________
