# Supplementary material for: Plasma-activated water as a wound rinse solution in patients with diabetes-related foot ulcers in two Australian hospitals: study protocol for a phase I double-blinded, randomised controlled trial
Source: BMJ Open. 2026 Jul 10;16(7):e118420. doi: 10.1136/bmjopen-2026-118420 (PMC13358267; doi:10.1136/bmjopen-2026-118420)
Supplement: online supplemental file 2 [file bmjopen-16-7-s002.pdf]

A Phase I randomised clinical trial of Plasma-Activated Water as a novel rinse agent for diabetes-related foot ulcers

Short Title: Plasma-Activated Water rinse for foot ulcers

HREC Number: TBA

Principal Investigator:	*Prof Robert Fitridge,* Consultant Vascular Surgeon, Vascular and Endovascular Service, Central Adelaide Local Health Network (CLAHN) [project design and recruitment]
Principal Scientists:	*A/Prof Katharina Richter,* Institute for Photonics and Advanced Sensing, The University of Adelaide [product preparation, project design]
	Dr Adrian Abdo, Department of Surgery, The University of Adelaide [product preparation, project design, and analysis]
Associate Investigators:	*Dr Guilherme Pena,* Senior Clinical Lecturer, The University of Adelaide [research design, report preparation]
	Dr Neil McMillan, Principal Medical Scientist, Vascular and Endovascular Service, CALHN [data management]
	Li Lao, Clinical Research Nurse, Vascular and Endovascular Service, CALHN [patient recruitment, sample collection]

Resourcing: Funding for Li Lao is provided to Dr Pena and Prof Fitridge via MRFF grant 2023153 to Richter/Abdo/Pena. Funding for investigational product and analyses are provided to Drs Richter and Abdo via same.

Conflicts of Interest: KR is a non-financial board member on the board of directors for RIBU Plasma Pty. Ltd. (Australia) and an inventor on patent WO/2024/013069 (Buske, C., & Richter, K. (2024). Process, apparatus and use of an apparatus for producing a plasma-activated liquid. Patent No. WO2024013069. World Intellectual Property Organization). KR will not analyse or interpret the data.

1 BACKGROUND AND RATIONALE

Introduction

Of the >1.2 million Australians living with diabetes, about 25% have a lifetime risk of developing a foot wound, >50% of those will develop infection, and >25% of those infections will become chronic/relapsing infections[1]. Patients with diabetes-related foot disease often present initially without clinically-relevant infection; however, even with gold-standard podiatric care and wound dressing solutions, many of these patients will develop diabetes-related foot infection that require hospitalisation. Infections that progress to necrosis and osteomyelitis often require surgical management leading to wound debridement or amputations with severe consequences to patient quality-of-life, morbidity, and mortality; and significant healthcare costs. Mortality after diabetes-related amputation is notoriously high with 70% at 5 years for all patients with diabetes and 74% at 2 years for those undergoing dialysis[1-4].

Pathogens typically reside in biofilms, which are clusters of microbes embedded in a protective slime. Biofilms hamper the efficacy of antimicrobial therapies by impeding drugs to reach pathogens and allowing bacteria to establish resistance[5]. The failure of best medical care with antibiotics, the lack of innovative products that are effective against biofilms, and the slow translation of promising treatments to human trials emphasise the urgent need to revolutionise infection control. We must find better treatments for multi-drug resistant (MDR) pathogens and biofilms while accelerating the translation of innovative therapies from bench to bedside. This is crucial if we want to lower morbidity and mortality linked to MDR infections, improve patients' quality-of-life, and reduce healthcare costs.

For this study, participants will have full wound standard-of-care provided by an experienced clinical trial wound nurse, with a wound rinse performed with one of two agents: saline control, or plasma-activated water (PAW). As described in the Investigator Brochure and below, this novel solution has the potential to be a rapid-acting wound cleaning agent that will kill bacteria in biofilm without engendering antibiotic-resistant organisms. As a first-in-human study of a product with a favourable safety profile, we expect this trial to place CALHN researchers and patients at the forefront of new infection control technologies.

Investigational Product Rationale

Cold plasma is an ionized gas consisting of excited ions, free electrons and radicals. When cold plasma is discharged in water it enriches the liquid with reactive oxygen and nitrogen species (RONS), which increases the redox potential and decreases the pH[6,7]. This creates an environment capable of effectively killing pathogens in vitro[8,9], while human cells remain unharmed due to internal mechanisms to neutralise RONS and pH. This makes cold plasma a potential breakthrough technology to improve infection control during wound management.

Plasma-activated water is safe to use and effective against MDR pathogens and biofilms in vitro. In collaboration with the company Plasmatreat, CI Richter and CI Abdo evaluated 5 types of PAW for antibacterial and antibiofilm activity against a range of MDR pathogens, including methicillin-resistant *Staphylococcus aureus* (MRSA) as one of the most difficult-to-treat superbugs that is highlighted on the WHO's global priority list to guide discovery, research and development of new treatments for MDR infections. CI Richter and CI Abdo identified one specific PAW type to show highest in vitro activity against MRSA planktonic

bacteria and biofilms, while being non-toxic to human skin cells (keratinocytes)[10]. Specifically, PAW exhibited >99.99% biofilm killing in microtiter assays, and reduced MRSA infection and accelerated skin wound healing in infected wounds in mice. Currently, CI's Richter and Abdo are investigating PAW in treating infected excisional wounds in pigs that most closely resemble human skin healing[11] to better translate PAW as an antimicrobial topical treatment for wounds..

To our knowledge, this is the first application of plasma-activated water in human wound care. There is one registered study for plasma-activated saline wound treatment (NCT05924867) by First Affiliated Hospital Xi'an Jiaotong University, but this study has not been updated since June 2023 and is yet to enrol participants as of June 2025.

Risk/Benefit Assessment

As a first-in-human trial, some unforeseen risks to participants are unavoidable; however, all existing preclinical data highlights minimal risk to human cells and wound healing. As a topical application, it is highly unlikely that any application of PAW can have systemic effects. No participant will have PAW applied until completion of an ongoing large-animal (pig) preclinical study demonstrates no ill effects.

As a novel agent applied to open wounds, it is possible that localised skin irritation may occur; the trial and care team will be aware of this as the most likely adverse reaction and tailor a care plan for cases where it eventuates in order to limit impact of irritation on the wound microenvironment and healing.

Preclinical mouse and pig models showed no signs of pain or discomfort on application of PAW, and our patients with DFU, who are predominantly neuropathic, have a decreased capacity to feel localised foot pain. If patients with incomplete loss of sensation do report any pain or discomfort from the rinse application, saline will immediately be applied to wash away PAW and the event reported as an adverse reaction.

As a Phase I study in patients without severe infection, benefits to participants are expected to be muted. We expect participants randomised to the experimental arm of this study to have reduced bacterial load and recurrent infection during study; preclinical data suggests that wound healing may also be improved for this group. Regardless, we expect participants in both groups to benefit from improved personalised care experienced by typical clinical trial participants.

2 STUDY OBJECTIVES AND OUTCOME MEASURES

Table 1 Study Objectives and Endpoints

**Primary Objectives**	**Primary Outcome Measures**
To assess the safety and tolerability of PAW up to 6 weeks after initiation of treatment.	The primary safety analysis will be performed based on data collected up to 6 weeks after initiation of treatment. Assessment of safety will be based on the Incidence and severity of adverse reactions, including but not limited to skin irritation or maceration.

Secondary Objectives	Secondary Outcome Measures
To evaluate the efficacy of PAW, at 12 and 18 weeks after initiation of treatment	Efficacy analysis will be performed based on data collected up to 18 weeks after initiation of treatment. Assessment of efficacy will be based on the following outcome measures of the study ulcer:<br>• Days to complete ulcer healing<br>• Presence and severity of infection<br>• Changes in wound quality based on ulcer assessment<br>• Quality of Life (Cardiff Wound Impact Schedule[12])

3 STUDY PLAN

Overall Study Design

This is a randomised, controlled, parallel-treatment study to investigate safety and tolerability of Plasma-activated Water (PAW) in adults with DFU.

Participants will be randomly allocated to one of the two treatment groups:

1. PAW
2. Control (sterile saline)

Participants will receive the appropriate rinse, applied to gauze as a temporary dressing, for ten minutes during a clinic appointment.

Design: A randomised, controlled, prospective trial. Participants will be patients with DFU.

Blinding: All clinical personnel and all participants will be blind to the assignment and treatment administered (double-blind). Only Dr Adrian Abdo will retain knowledge of treatment based on preparation of treatment and linkage on a spreadsheet with study ID. This spreadsheet will be used to match for study safety and efficacy on end of study, and/or once an SUSAR has been confirmed.

Screening and Randomisation: Participants who provide consent to participate in the study and meet all eligibility criteria and no exclusion criteria will be randomly assigned to one of the two groups.

Treatment and Evaluation Period: Participants will attend a total of 12 scheduled visits over 6 weeks, plus follow-up wound review visits fortnightly until week 12 and final visit at week 18.

A study flow diagram is provided in Figure 1.

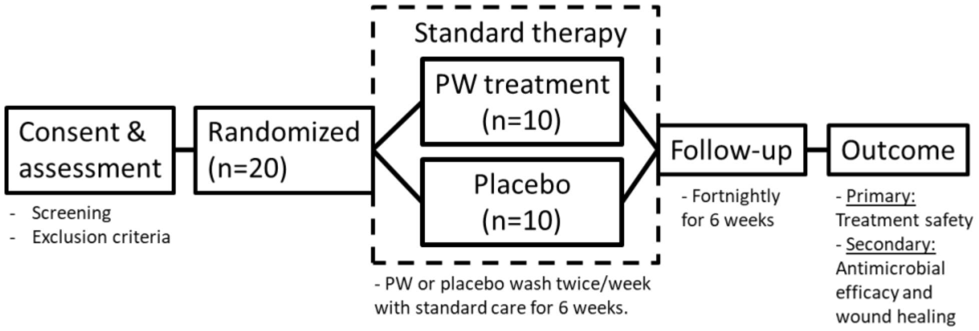

Figure 1 Study Flow Diagram

Number of Participants

The study will aim to recruit 10 participants per study group (approximately 20 participants in total).

Study Period/Duration of Participation

The study will involve:

- A treatment (six weeks) with follow-up for six weeks (fortnightly) and at six weeks following.

Total: approximately 18 weeks per participant.

The overall study period is expected to be September 2025 to August 2026.

Recruitment of Participants

Participants will be recruited from the multidisciplinary foot clinics (MDF) and podiatry outpatient clinics at the RAH and TQEH. Patients in these services are well known to clinical staff and the investigators. Potential participants will be identified by clinical staff, and initial approach about participating in the trial will be performed by the patient's attending clinician (vascular, nursing, or podiatry). Information and consent will be provided by a clinical study investigator.

Participants will be given two weeks to consider participation, but will also be allowed to screen in earlier if they choose and are deemed by the investigator to have adequately considered their options. Participants will be specifically reminded during this process that participation or non-participation will not impact their care, and that they are allowed to withdraw from the study at any time.

Inclusion Criteria

Prospective participants must meet all the following inclusion criteria to be eligible to participate in the study:

1. Adults at least 18 years of age.
2. Have a current diagnosis of diabetes mellitus (DM) characterized by at least one of the following:
    - currently under DM medication treatment

- with HbA1c > 6.5%
- with fasting plasma glucose > 7.0 mmol/L
- with plasma glucose > 11.1 mmol/L in the two-hour 75-gram oral glucose tolerance test (OGTT).

3. With at least one cutaneous ulcer on the foot, which meets all the following criteria:

- area between 2 and 20 cm^2 at the time of enrolment.
- has been under the standard care of the Investigator for at least 4 weeks.

NOTE: if a participant has two or more ulcers that meet the inclusion criteria, only one ulcer will be selected as the study ulcer. The ulcer selected should be the one with the largest area. The other ulcers will be treated by standard care.

4. With ankle brachial index (ABI) ≥ 0.4 and/or toe pressure >30 mmHg on the limb with the study ulcer.

5. The participant has a life expectancy of at least 6 months as determined by the Investigator based on a combination of medical history, physical examination, vital signs, clinical laboratory tests, and other risk stratification assessments.

6. Able to follow the Investigator's instruction on ulcer care (in the opinion of the Investigator).

7. Able to provide written informed consent. If cognitive impairment is suspected but the patient otherwise meets criteria, consent will only be obtained with a friend or family member signing off as witness.

Exclusion Criteria

If prospective participants meet any of the following exclusion criteria, they are ineligible to participate in the study:

1. Planned for revascularisation (open or endovascular) or any major or minor amputation of the index leg within the next 3 months.
2. Active moderate-to-severe infection in the study ulcer, or treatment with intravenous antibiotics within the past two weeks.
3. Any sign (clinical, radiographic) of osteomyelitis associated with the study ulcer.
4. Treatment with systemic immunosuppressants within 90 days of screening.
5. Active malignancy or history of malignancy within five years prior to screening (except for a past history of basal or squamous cell carcinomas).
6. Known history of HIV infection.
7. Received any investigational research agent within 60 days or within five half-lives of the last treatment (if the half-life of the investigational agent is known to be longer than 12 days) prior to the planned initiation of study treatment.
8. With any clinical condition or significant concurrent disease judged by the Investigator to complicate the evaluation of the trial treatment.

Method of Assigning Participants to Treatment Group

Participants will be randomly assigned to one of two treatment groups once written informed consent has been obtained and eligibility established.

A participant identification (ID) number will be allocated to each participant who provides informed consent, so that participants can be identified without making assumptions about their subsequent eligibility for the study. Participants will be allocated to sequential, ascending 2-digit ID numbers (e.g, 01, 02, 03), which will provide a unique identifier. The participant will retain the same ID number for the duration of the study.

If a participant fails screening or discontinues from the study, the participant's ID number will not be reused.

Treatments will be prepared according to a list of study IDs and assignments created from random.org, with the spreadsheet of assignment retained by Dr Adrian Abdo. At no time will Dr Abdo have knowledge of patient identity or clinical information for any patient; and at no time will Li Lao or Dr Guilherme Pena know the assignment of a patient to PAW or control.

Withdrawal of Participants from Study

Participants can terminate their study participation at any time and without giving a reason, without prejudice to further treatment. Participants who discontinue from the study will be asked about the reason(s) for their discontinuation only to determine the presence of any AEs. If possible and willing, they will be seen and assessed by an Investigator and have a final study visit. AEs should be followed up until resolved or stable and determined to be chronic.

Participants who withdraw from the study prior to the final scheduled visit will have the final visit procedures conducted at the time of withdrawal, if possible. If a participant withdraws due to the need for amputation of the foot/limb with the study ulcer, they will be asked to have the final visit procedures prior to the amputation, if possible.

If a participant withdraws due to the study wound being completely healed, they should have the final visit procedures.

On withdrawal, participants will have the opportunity to request that their existing data and samples be expunged from the study, to the extent it is possible to do so.

The Principal Investigator can exclude a participant from continuing in the study. Possible reasons for discontinuing a participant may include:

- Participant withdrawal of consent
- Inability to tolerate the study treatment
- Any unacceptable AEs, in the judgement of the Principal Investigator
- Participant's non-adherence to the protocol
- The use of another investigational research agent prior to Week 6 has been deemed necessary
- Termination of the study for administrative, financial, or other reasons.

Replacement of Participants

Approximately 20 participants will be recruited in total. Additional participants may be enrolled into the study at the discretion of the Principal Investigator to ensure at least 10 evaluable participants per treatment group. Participants who withdraw due to adverse events (AEs) will not be replaced.

4 STUDY PROCEDURES

Participant Information and Informed Consent

The Investigator must provide adequate information regarding the study conduct and obtain written informed consent from the participant before any tests or investigations outlined in the study protocol are carried out.

The participant will be given time to read and understand the Participant Information and Consent Form (PICF), and have any questions answered. They may wish to take the PICF and consider it further, or to discuss it with their family before signing.

The PICF must be personally signed and dated by both the Investigator obtaining consent and the participant.

Overview of Study Visits

Study procedures should be performed at relevant study visits in accordance with the schedule of procedures outlined in Table 1. The Principal Investigator or other study doctor will be present for study treatment at screening and at the week two visit at minimum to ensure patient safety and observe for AEs.

Participants will only be enrolled and randomised to study treatment if they meet all eligibility criteria, and none of the exclusion criteria.

Study visits will be arranged at Royal Adelaide Hospital and/or The Queen Elizabeth Hospital, aligning with patient clinical visit scheduling where possible to minimise inconvenience to participants. A window of plus or minus three days is permitted for Visits 1 to 6, and a window of plus or minus seven days is permitted for follow-up Visits 7-10. Study visits via home visit are permitted for Visits 3-10.

Data collection

Demographic data will include:

- Age
- Gender
- Ethnicity
- Race
- If the participant holds a healthcare card

Medical history will include (to the extent that details are known):

- Date of diabetes diagnosis
- Diabetes type

- History of peripheral artery disease and related conditions
- Details of peripheral neuropathy
- Date of ulcer onset
- Details of previous ulcer assessments
- Details of previous interventions/treatments relevant to DFU
- History of cardiovascular events
- History of renal disease with or without dialysis
- Use of immunosuppression
- Smoking status current / past

Study period	Screening	Treatment and Evaluation									
Visit number		1	2	3	4	5	6	7	8	9	10
Study Week		1<br>(weekly)	2	3	4	5	6	8<br>*(Fortnightly follow-up)*	10	12	18<br>*(Final visit)*
Study treatment		X	X	X	X	X	X				
Study day		0	7	14	28	35	42	56	70	84	168
Window days		+/- 3	+/- 3	+/- 3	+/- 3	+/- 3	+/- 3	+/- 7	+/- 7	+/- 7	+/- 7
Informed consent	X										
Eligibility review	X	X									
Demographics	X										
Medical history	X										
Physical assessment	X	Symptom-directed	Symptom-directed	Symptom-directed	Symptom-directed	Symptom-directed	Symptom-directed	Symptom-directed	Symptom-directed	Symptom-directed	Symptom-directed
Vital signs	X	X	X	X	X	X	X	X	X	X	X

Any medications or dressings/ulcer treatments used by participants in the 21 days prior to Day 0 and or/during the study will be documented in the participant notes. This record should include the treatment name, the dose and frequency, form and route of administration, the start and stop date of administration, and the indication for which the treatment is being used.

Identification of the Study Ulcer

If the participant has two or more diabetes-related foot ulcers that meet the eligibility criteria, only one will be selected as the 'study ulcer' (i.e., the ulcer that will be treated with study treatment and used as the basis of outcome measures). The ulcer selected should be the one with the largest area. Other ulcers will be treated by standard of care throughout the study.

The location of the study ulcer will be recorded as the foot (left or right), surface (plantar, dorsal, medial, or lateral), and area (forefoot, midfoot, hindfoot). Toe locations will be recorded from first (hallux) to fifth (the outermost toe).

Safety and Tolerability Assessments

Safety will be assessed by physical examinations, vital signs, laboratory tests, wound characteristics and recording of AEs.

Physical Examinations

Physical examinations will include:

- Height (at screening only).
- Weight.
- A symptom-directed physical examination, including thorough assessment of the index ulcer and leg.
- ABI and toe pressure (screening only).
- New or worsening clinically significant abnormalities will be reported as an AE.

Vital Signs

Prior to first study treatment, vital signs will be measured after the participant has been sitting or supine for five minutes and will include pressure on both arms, pulse rate, respiratory rate and body temperature.

Laboratory Tests

Blood samples will be taken twice throughout the course of the study for safety assessments – at screening and at two weeks - plus as clinically indicated. These are part of routine clinical care in this cohort. The following specific tests will be performed:

- Haematology: Haemoglobin, haematocrit, red blood cell (RBC) count, white blood cell (WBC) count with differential, and platelet count.

- Clinical chemistry: Sodium, potassium, calcium, serum albumin, total protein, gamma glutamyl transferase (GGT), aspartate aminotransferase (AST), alanine aminotransferase (ALT), alkaline phosphatase (ALP), total bilirubin, lactate dehydrogenase (LDH), glucose and creatinine. Creatinine clearance is calculated based on Cockcroft-Gault equation, not raw serum creatinine.
- HbA1c will be performed at Screening (unless recent result is available).

Wound characteristics assessments

At each study visit, a clinical Investigator will assess the characteristics of the study ulcer using the Monash Wound Assessment used throughout our clinical practice:

- Location
- Type – neuropathic, ischaemic, neuro-ischaemic
- Size - length, width, depth (post debridement).
- Involvement of deep structures, sinus tracts, tunnelling
- Wound bed tissue type
- Signs of infection
- Exudate - type and quantity
- Odour
- Pain

5 ADVERSE EVENTS

Participants will be questioned and monitored throughout the study with regard to any adverse events (AEs) they may have experienced. An AE can therefore be any unfavourable and unintended sign (including an abnormal laboratory finding), symptom, or disease temporally associated with the use of a medicinal product, regardless of relationship to the medicinal (investigational) product.

Determinations of severity and causality of any AE can only be determined by a clinical Investigator without awareness of whether the treatment used is PAW or control.

An AE shall be classified as serious (a Serious Adverse Event, or SAE) if, in the opinion of either the Investigator or the Sponsor, it:

- Results in death.
- Is life-threatening, i.e., in which the participant was at risk of death at the time of the event.
- Results in permanent impairment of a body structure or body function
- Requires in-patient hospitalisation or prolongation of existing hospitalisation.
- Results in persistent or significant disability/incapacity.

Important medical events that are not immediately life-threatening and that do not result in death or hospitalisation may still be considered serious if, in the opinion of the Investigator, they jeopardise the participant or require intervention to prevent one of the other outcomes listed above. Such events should also be treated as SAEs.

Assessment of Causality

The Investigator must assess the causality of each AE and SAE. Assessing causality involves consideration of whether there is a reasonable possibility that the event may have been caused by the treatment.

- **Not related:** AE for which there is evidence of another explanation, e.g. the AE is obviously explained by the participant's disease(s), is in accordance with the known effect of a concomitant medication or has occurred prior to first administration of study treatment.
- **Unlikely related:** AE with a time to study treatment administration that makes a relationship improbable (but not impossible), and disease or other drugs provide plausible explanations.
- **Possibly related:** AE with a reasonable time relationship to study treatment administration, but which could also be explained by disease or other drugs.
- **Probably related:** AE with reasonable time relationship to study treatment administration that is unlikely to be attributed to disease or other drugs.
- **Definitely related:** AE with plausible time relationship to study treatment administration, which cannot be reasonably explained by the known characteristics of the participant's clinical state, environmental factors, or other modes of therapy administered to the participant.

All AEs must be documented by the Investigator, regardless of causality.

Recording of Adverse Events

All AEs that occur from the time of informed consent until the participant completes or withdraws from the study will be recorded in REDCap.

SAEs deemed possibly, probably or definitely related to study treatment occurring after the participant completes the study will also be reported to the Sponsor, or delegate, if the Investigator becomes aware of them.

It is preferable that AEs are reported as diagnoses if one is able to be made, rather than individual signs and symptoms. The AE description, onset and resolution dates, severity, causality and outcome must be recorded, as well as any actions taken.

Unless a diagnosis is made, or signs and symptoms are present, laboratory values or vital signs abnormalities should only be reported as AEs if they cause the participant to discontinue from the study, the Investigator feels it is clinically significant, or they meet a criterion for a SAE.

If an AE leads to premature discontinuation of the study, the appropriate pages of REDCap must be completed.

Reporting of Serious Adverse Events

Investigators and other site personnel must report SAEs to the Sponsor or designee within 24 hours of becoming aware of the SAE, regardless of causality.

Follow-up information on SAEs must also be reported by the investigational site within the same time frame. If a non-serious AE becomes serious, this and other relevant follow-up information must also be provided within 24 hours.

All SAEs will be recorded in the participant records and REDCap.

Follow-Up of Adverse Events and Serious Adverse Events

All AEs and all SAEs must be followed by the Investigator until resolution, until the AE stabilises or is recognised as a permanent condition by the Investigator, or until the participant is lost to follow-up, whichever comes first. Follow-up investigations may be necessary according to the Investigator's medical judgement.

6 EFFICACY ASSESSMENT

Ulcer Healing Assessments

The Investigator/nurse will inspect the study ulcer at each site visit for physical (rate of healing) characteristics.

An ulcer will be considered completely healed if there is 100% reepithelialisation of the ulcer surface with no trace of exudate or drainage, as determined by the Investigator. The date of complete healing will be recorded as the date of the first assessment of 100% reepithelialization.

Note: if a participant withdraws from the study because their study wound is completely healed, they should complete Week 24 assessments.

Ulcer Infection

Ulcer swabs will be taken as per Levine technique before and after application of rinse (PAW or control) at Weeks 1, 3, and 6. Samples will be processed by Dr Adrian Abdo's team at the Basil Hetzel Institute.

Ulcer Pain

Ulcer pain will be assessed using an 11-point Numeric Rating Scale (NRS). Participants will be asked to verbally rate the pain intensity in their study ulcer over the previous 24 hours as an integer from 0 to 10 (0 = no pain, 10 = worst possible pain). The majority of patients likely to be included in the study have peripheral neuropathy and are unlikely to feel pain at site; pain scores will be recorded nonetheless.

Contact Dermatitis assessment

As the most likely adverse reaction to study treatment, all participants will be specifically monitored for contact dermatitis at study site throughout each study visit.

Quality of Life questionnaire

Participants will be asked to complete a questionnaire to assess health-related quality of life (The Cardiff Wound Impact Schedule), as below (Figure 2). Use for academic projects is provided without charge.

Physical symptoms and daily living

Have you experienced any of the following during the past week?

	Not at all/ Not applicable	Seldom	Sometimes	Frequently	Always
Disturbed sleep	☐	☐	☐	☐	☐

Physical symptoms and daily living

How stressful has this experience been for you?

	Not at all/ Not applicable	Slightly	Moderately	Quite a bit	Very
Disturbed sleep	☐	☐	☐	☐	☐

Social life

Have you experienced any of the following during the past week?

	Not at all/ Not applicable	Seldom	Sometimes	Frequently	Always
Difficulty getting out and about	☐	☐	☐	☐	☐

Social life

How stressful has this experience been for you?

	Not at all/ Not applicable	Slightly	Moderately	Quite a bit	Very
Difficulty getting out and about	☐	☐	☐	☐	☐

Well-being

To what extent do you agree/disagree with the following statements?

	Strongly disagree	Disagree	Not sure	Agree	Strongly agree
I feel anxious about my wound(s)	☐	☐	☐	☐	☐

Figure 2. The Cardiff Wound Impact Schedule.

7 STUDY TREATMENTS

Description of the Investigational Medicinal Product

Description of Standard Care

The standard care will follow the best practice for the type, severity and stage of ulcer for each participant. This will be at the investigator's discretion. Details of the standard care will be recorded.

IP Storage, Handling and Preparation

IP will be prepared by Dr Adrian Abdo in accordance with the Investigator Brochure. Bottles of IP/saline will be stored in the Basil Hetzel Institute building clinical trial room 2, labelled with study ID. Bottles will be used to soak gauze for rinsing the wound of each participant according to their study ID and the protocol.

IP Administration and Post-Dose Monitoring

Only participants enrolled in the study may receive study treatment. The Principal Investigator (PI) or authorised designee will ensure that the study treatment at site is safely handled in compliance with requirements. The PI is responsible for ensuring that the study treatment is dispensed in accordance with the protocol and only to participants enrolled in the study.

Criteria for Postponing or Stopping Study Treatment

Prior to the Application of Treatment

The treatment should not be applied if, in the opinion of the Investigator, there is a risk that the participant will be unable to tolerate treatment for any reason.

Treatment applications should be deferred if probable ulcer infection is noted. If treatment is deferred the patient can re-enter the trial at the point at which they left once the problem settles and at the Investigator's discretion.

Under these circumstances, at the discretion of the Investigator, study treatment may be postponed and initiated at a later time, provided that:

- The problem that resulted in postponement of the study treatment resolves, and is not considered by the Investigator to be likely to recur; AND
- The relevant visit is still expected to be completed within the stipulated visit window.

Alternatively, the participant may be withdrawn from the study.

Application of Treatment

The treating clinician will identify the patient by their study ID and check that they have the appropriate ID-labelled treatment. All wound dressings should be removed, and if standard of care requires debridement of the wound, that debridement will be done prior to the application of study treatment.

A piece of sterile gauze is soaked in product, and applied to the wound for ten minutes. Other study or clinical procedures can be performed during the ten-minute period.

After the Application of Treatment

Once dressing is applied, its application should be immediately removed if:

- in the opinion of the Investigator, a participant exhibits signs or symptoms of shock or an allergic reaction; OR
- in the opinion of the Investigator, a participant exhibits any other unacceptable AE that warrant its removal; OR
- if the participant requests its removal.

When a rinse dressing is removed, it should not be reapplied, and the time and date of removal should be recorded.

Accountability of Study Supplies

All material supplied is for use only in this clinical study and should not be used for any other purpose. Used agents will be discarded by clinical staff after being removed from the ulcer.

Blinding

Concomitant Treatments

All participants will receive standard ulcer care.

Aside from treatment of the study ulcer, participants may continue to take their usual medications or other medications deemed necessary by the Investigator or other prescribing healthcare practitioner, with the exception of other investigational research agents and/or immunosuppressive agents. Investigational research agents are defined as agents that have not been granted a marketing authorisation (or equivalent) for the relevant indication in the relevant jurisdiction.

If the Investigator or other prescribing healthcare practitioner deems it necessary to use another investigational research agent, and/or an immunosuppressive agent, then the participant should be withdrawn from the study prior to commencement of the new treatment if possible.

8 DATA AND SAMPLE MANAGEMENT

Data collection and entry into REDCap will be completed by authorised SA Health personnel designated by the Investigator and named on this protocol. Appropriate training and security measures will be completed with the Investigator and all authorised study site personnel prior to the study being initiated and any data being entered into the system for any study participants. Data will be stored in an identifiable format (with patient URN). Data will only be downloaded and shared with University investigators and biostatistician in de-identified format; patient URN will be marked as an identifier in REDCap and removed from data exports. Procedure dates will be date-shifted in REDCap to maintain confidentiality. Identifiable data will not be accessed by non-CALHN employees. If data need to be re-identified, they can be matched by CALHN investigator against their study ID.

REDCap is a data entry form and should not constitute the original, or source document. Source documents are all documents used by the Investigator or hospital that relate to the participant's medical history, that verify the existence of the participant, the inclusion and exclusion criteria and all records covering the participant's participation in the study. They include, but are not limited to, laboratory reports, treatment dispensing records, hospital records, participant files, etc.

All participant samples will be stored in re-identifiable format by study ID at the Basil Hetzel Institute for Translational Health Research. These samples will be owned by the University of Adelaide, and staff and students of the University will analyse samples as permitted by individual participant consent. Samples will be stored based on the logged consent options defined by the participant: until the completion of the study, or for up to 5 years to allow for other relevant studies to be performed. Dr Adrian Abdo or his delegate will be responsible for annotation of samples for storage periods, custodianship and management, and destruction of samples on schedule as biological waste.

9 STATISTICAL ANALYSIS

The statistical analysis principles described below will be supplemented by a statistical analysis plan (SAP), which will be finalised before the database is locked. Any changes to the statistical plans will be described and justified in the clinical study report (CSR).

The SAP will describe procedures for accounting for missing, unused, and spurious data. This section is a summary of the planned statistical analyses of the primary and secondary endpoints.

All analyses, summaries, and listings will be performed using SAS® software (version 9.4 or higher).

The following descriptive statistics will be used as applicable to summarise the study data, unless otherwise specified:

- Continuous variables: sample size [n], mean, standard deviation [SD], median, minimum [min], and maximum [max].
- Categorical variables: frequencies and percentages.

Individual participant data will be presented in listings.

Sample Size Assumptions

The sample size for this study is approximately 20 participants with DFU, with approximately 10 participants in each of the two treatment groups.

As this is a Phase 0/1 study, no formal sample size has been calculated. Ten participants per group is considered appropriate for a study of this nature.

Demographics and Baseline Data

Participant enrolment and disposition will be summarised for participants enrolled into the study and in each analysis set. The number of participants prematurely discontinuing from the study, along with the reason for early discontinuation will also be summarised.

Demographic and baseline data recorded at screening and prior to dosing will be summarised for all participants.

Prior medications are those medications taken on or before the first investigational product administration. Concomitant medications are medications that are taken after the first investigational product administration. Medications taken on the same day as investigational product administration will be considered as prior and concomitant medications.

Efficacy Analyses

Efficacy indicators will be summarised descriptively with incidence rates or standard descriptive methods, as appropriate.

Efficacy analysis will be based on data collected up to 12 weeks after initiation of treatment. Assessment of efficacy will be based on the following outcome measures of the study ulcer:

- Days to complete ulcer healing
- Changes in ulcer quality (e.g., infection, signs, pain) on the Monash Diabetes Foot Assessment tool

Safety Data

Adverse events will be coded using the Medical Dictionary for Regulatory Activities (MedDRA). For each study treatment, number of participants, percentage of participants in the treatment group and numbers of TEAEs will be tabulated by system organ class and preferred term.

The study treatment information will be listed. The actual number of dressings applied will be summarised.

For the summaries of AEs, participants who experience the same AE (in terms of the MedDRA system organ class and preferred term) more than once will only be counted once for that event in the number of participants. Categorical summaries will include the

frequency, incidences (one per participant) and percentages of events for participants and will be displayed by treatment group.

Clinical Laboratory Evaluations

Laboratory data (haematology, clinical chemistry, HbA1c) will be summarised for each treatment group using descriptive statistics at each protocol scheduled visit for actual values and changes from baseline.

Abnormal laboratory values will be flagged and will be identified in the listings. Microscopy data, if available, will be listed.

The incidence of treatment-emergent abnormal laboratory findings will be summarised using frequency counts and percentages for each treatment group and listed separately.

Vital Signs Analysis

Vital signs (including height, weight and BMI) will be summarised for each treatment group using descriptive statistics at each protocol scheduled time point for actual values and change from baseline.

Other Safety Measures

Physical Examination findings will be listed only.

10 STUDY MANAGEMENT

Quality Control and Quality Assurance

All study tasks will be performed in accordance with applicable regulations, ICH Good Clinical Practice (GCP), and study site Standard Operating Procedures (SOPs).

During the study, CALHN Research Office staff may regularly visit the site to monitor and confirm protocol, regulatory and ethical adherence, confirm data accuracy and provide information and support as needed.

The PI agrees to allow designated CALHN personnel direct access to all relevant documents, including electronic medical records, and to allocate his time and the time of his staff to the CRA to discuss findings and any relevant issues.

Data Protection

The PICF will explain that study data will be stored in a computer database, maintaining confidentiality in accordance with national data legislation. In this database participants will be identified by participant ID number only. The PICF will also explain that for data verification purposes, authorised representatives of the Sponsor, regulatory authorities, IECs or sites may require direct access to parts of the hospital or site records relevant to the study, including personal participant information.

Archiving

The Investigator is responsible for the archiving of the study records for their site. Study records include the participant files as well as the source data, the Investigator Site File,

pharmacy records, and other study documents. Study records must be archived for at least 15 years. After 15 years, secure destruction of data (shredding of paper copies, secure deletion of REDCap resources) will be directed by Dr Neil McMillan or delegate.

If the Investigator leaves the investigational site for any reason, the responsibility for all study related records must be transferred to another person at site.

Publication Policy

All study staff will be named on any publications arising from the research. Study participants will be supported to receive copies of the publication(s) should they wish.

11 REFERENCES

1. Armstrong DG, et al. 2017. Diabetic Foot Ulcers and Their Recurrence. N Engl J Med, 376(24):2367-2375.

2. Lavery LA, et al. 2010. Impact of chronic kidney disease on survival after amputation in individuals with diabetes. Diabetes Care, 33(11):2365-9.

3. Lazzarini PA, et al. 2017. The silent overall burden of foot disease in a representative hospitalised population. Int Wound J, 14(4):716-728.

4. Jeffcoate W, Boyko EJ, Game F, Cowled P, Senneville E, Fitridge R. Causes, prevention, and management of diabetes-related foot ulcers. The Lancet Diabetes & Endocrinology.

5. Liu, H.Y., Prentice, E.L. & Webber, M.A. 2024. Mechanisms of antimicrobial resistance in biofilms. npj Antimicrob Resist 2:27.

6. Thirumdas R, et al. 2018. Plasma activated water: Chemistry, physico-chemical properties, applications in food and agriculture. Trends Food Sci Technol, 77:21-31.

7. Wang Q, Salvi D. 2021. Evaluation of plasma-activated water as a novel disinfectant: Effectiveness on Escherichia coli and Listeria innocua, physicochemical properties, and storage stability. LWT – Food Sci Technol, 149:111847.

8. Laurita R, et al. 2015. Chemical analysis of reactive species and antimicrobial activity of water treated by nanosecond pulsed DBD air plasma. Clin Plasma Med, 3(2):53-61.

9. Zhang Q, et al. 2016. Sterilization efficiency of a novel electrochemical disinfectant against Staphylococcus aureus. Environ Sci Technol, 50(6):3184-3192.

10. Abdo A, Schmitt-John T, Richter K. 2021. Plasma-activated water as a new weapon against multidrug-resistant bacteria. ECCMID 2021, EMJ Microbiol Infect Dis, 2(1):38-39.

11. Summerfield, A., F. Meurens, and M.E. Ricklin, The immunology of the porcine skin and its value as a model for human skin. Mol Immunol, 2015. 66(1): p. 14-21.

12. Price PE and Harding KG (2004) The Cardiff Wound Impact Schedule: the development of a condition specific questionnaire to assess health-related quality of life in patients with chronic wounds. International Wound Journal 1(1):10-17.
