# Supplementary material for: Plasma-activated water as a wound rinse solution in patients with diabetes-related foot ulcers in two Australian hospitals: study protocol for a phase I double-blinded, randomised controlled trial
Source: BMJ Open. 2026 Jul 10;16(7):e118420. doi: 10.1136/bmjopen-2026-118420 (PMC13358267; doi:10.1136/bmjopen-2026-118420)
Supplement: Supplementary file 3 [file bmjopen-2026-118420.reviewer_comments.pdf]

PEER REVIEW HISTORY

BMJ Open publishes all reviews undertaken for accepted manuscripts. Reviewers are asked to complete a checklist review form and are provided with free text boxes to elaborate on their assessment. These free text comments are reproduced below.

ARTICLE DETAILS

Title (Provisional)

Study protocol for a phase I double-blinded, randomized controlled trial of plasma-activated water as a wound rinse solution in patients with diabetes-related foot ulcers in two Australian hospitals

Authors

Abdo, Adrian I.; Mcmillan, Neil; Lao, Li; Pena, Guilherme N; Fitridge, Robert; Richter, Katharina

---

VERSION 1 - REVIEW

**Reviewer**	**1**
**Name**	**Heald, Adrian**
**Affiliation**	**Salford Royal Hospitals NHS Trust, Endocrinology and Diabetes**
**Date**	**27-Apr-2026**
**COI**	**None**

Overview

Diabetes foot ulceration is a very significant problem for the affected individual and is also a marker of major systemic consequences of diabetes.

Diabetes foot ulceration often requires a prolonged course of treatment and may be recurrent. Any intervention that can decrease wound healing time is of immense value. The protocol as written is clear as are the study aims. I note that ethical approval has already been granted. Also, PPRE has been performed. I note that data management is appropriately organised.

Specific points

With regard to the Introduction, I suggest that a relevant paper on the diabetes ulcer related mortality rate be included (Schofield H, Haycocks S et al. Diabet Med. 2021 Oct;38(10):e14568). There are major socioeconomic determinants of diabetes foot ulceration, which also impact on more adverse outcomes in those with a history of diabetes

foot ulceration, including higher mortality rate. Please can this relevant paper be cited (Anderson SG, Shoo H et al. Diabetologia. 2018 Apr;61(4):959-967).

The potential for fungal infection as factor driving a prolonged foot ulcer healing time or recurrent foot ulcereation could be included (Heald AH, O'Halloran DJ et al. Diabet Med. 2001; (7):567-72

In the methodology I would suggest adding the sending of a tissue swab for mycology (fungal culture) as part of the microbiological work up

---

**Reviewer**	**2**
**Name**	**Zhao, Yang**
**Affiliation**	**China Academy of Chinese Medical Sciences Xiyuan Hospital**
**Date**	**22-May-2026**
**COI**	**None**

This is a well-designed, ethically sound Phase I randomised controlled trial protocol with clear clinical relevance and innovation. The double-blind design, incorporation of the intervention into existing clinical workflows, and appropriate safety focus are strengths. However, several methodological limitations should be addressed before the protocol is accepted for publication. Major concerns include the very small sample size (n=20), insufficient power for efficacy endpoints, and relatively short follow-up period for chronic diabetic foot ulcers. The authors should explicitly frame this trial as a pilot safety study and refrain from any claims of superiority of plasma-activated water (PAW) over saline in promoting healing or reducing infection rates.

Specific Comments:

1. Risk of unblinding due to sensation

PAW may cause a stinging sensation or discomfort, which could lead patients or nurses to guess their group allocation. Recommendation: Record any instances of accidental unblinding and conduct a sensitivity analysis; An assessment of blinding success at the end of the trial (e.g., asking participants and the treating nurse to guess the assigned treatment).

2. “Standard treatment” is not clearly defined

The protocol repeatedly refers to “standard care” but does not specify what it includes. The authors should provide an explicit definition, the specific components of standard treatment (such as the frequency of debridement, methods of pressure relief, and blood glucose management) should be clearly defined

3. The ulcer area is too large

Inclusion requires an ulcer area between 2 cm² and 20 cm². This broad range introduces substantial heterogeneity in healing potential and infection risk. It is recommended that this be reduced, or that the baseline area be corrected in the analysis.

VERSION 1 - AUTHOR RESPONSE

Reviewer: 1
Dr. Adrian Heald, Salford Royal Hospitals NHS Trust, The University of Manchester
Comments to the Author:
Overview
Diabetes foot ulceration is a very significant problem for the affected individual and is also a marker of major systemic consequences of diabetes.

Diabetes foot ulceration often requires a prolonged course of treatment and may be recurrent. Any intervention that can decrease wound healing time is of immense value. The protocol as written is clear as are the study aims. I note that ethical approval has already been granted. Also, PPRE has been performed. I note that data management is appropriately organised.

Thank you for your approving comments. We tried to make our protocol as clear and transparent as we could.

Specific points

With regard to the Introduction, I suggest that a relevant paper on the diabetes ulcer related mortality rate be included (Schofield H, Haycocks S et al. Diabet Med. 2021 Oct;38(10):e14568). There are major socioeconomic determinants of diabetes foot ulceration, which also impact on more adverse outcomes in those with a history of diabetes foot ulceration, including higher mortality rate. Please can this relevant paper be cited (Anderson SG, Shoo H et al. Diabetologia. 2018 Apr;61(4):959-967).

We appreciate the suggestion and read the Schofield et al. (2021) paper carefully. While we recognise its value, we felt it did not closely align with our specific focus on morbidity and mortality attributable to DFU-related amputation, as the paper follows all-cause mortality in a T1D/T2D cohort without clear attribution to foot ulceration. We have therefore opted not to include it, but thank the reviewer for bringing it to our attention.

Regarding Anderson et al, 2018, there is valuable mortality data for the formation of foot ulcers in the Cox regression model, which showed a significant hazard ratio of 1.5 for mortality associated with foot ulcers. As such, we have added this to the first paragraph of the introduction:

"In people with diabetes, just the presence of a foot ulcer has been shown to increase risk of mortality by nearly 50% compared to diabetes without a foot ulcer.[2] "

The potential for fungal infection as factor driving a prolonged foot ulcer healing time or recurrent foot ulcereation could be included (Heald AH, O'Halloran DJ et al. Diabet Med. 2001; (7):567-72

Thank you for the suggestion, for the sake of keeping the manuscript focussed, and because this manuscript is describing a protocol, we do not intend to broaden the scope of our introduction to describe all factors of DFU, as there are a multitude to go through. Instead, we opted to:

(i) keep the introduction focussed on defining DFU and its detrimental outcomes (not the causes/cofactors/confounders) in terms of morbidity and mortality,
(ii) describe the treatment challenges as brought forward by the IWDGF guidelines and recommendations to rationalise our study and protocol, and;
(iii) Provide the relevant background information on PAW and the scientific justification for going towards clinical trial, which the protocol needs.

When the trial is completed and results analysed, the above reference and others can be cited in relation to the microbiology results obtained from the trial for discussion and comparison.

In the methodology I would suggest adding the sending of a tissue swab for mycology (fungal culture) as part of the microbiological work up.
Thank you for the suggestion, and yes, we agree. Our main research focus is on bacterial microbiology, as clearly reflected in our protocol, but incidence and attributable mortality of Candidiasis infection is high [1], and fungal colonisation (if not necessarily infection) are also frequent in patients with DFU [2]. We have purchased specialised agar for growing fungi, particularly for differentiating Candida species which are the overwhelming fungal majority colonising wounds, but this occurred after submission of the first manuscript. So, we have taken this opportunity to include details about this in the revised manuscript:

Under "*Study design*" we have included microbial swabs will also be taken to determine "fungal load."

Under "*Clinical laboratory measurements*" we have included the following at the end:
"Microbiological swabs will then be stored long term at -80°C in saline with 25% glycerol: 50% tryptone soya broth for biobanking. These biobank samples will be used for bacterial and fungal identification and isolation using selective and differential chromogenic agars. Bacterial and fungal isolates will be biobanked and tested for susceptibility to antimicrobials and PAW *in vitro*."

Reviewer: 2
Dr. Yang Zhao, China Academy of Chinese Medical Sciences Xiyuan Hospital
Comments to the Author:
This is a well-designed, ethically sound Phase I randomised controlled trial protocol with clear clinical relevance and innovation. The double-blind design, incorporation of the intervention into existing clinical workflows, and appropriate safety focus are strengths. However, several methodological limitations should be addressed before the protocol is accepted for publication. Major concerns include the very small sample size (n=20), insufficient power for efficacy endpoints, and relatively short follow-up period for chronic diabetic foot ulcers. The authors should explicitly frame this trial as a pilot safety study and refrain from any claims of superiority of plasma-activated water (PAW) over saline in promoting healing or reducing infection rates.

Thank you for your approving comments.

This is small trial, but with 20 participants followed for several months, it still fits the model of a phase I trial. We acknowledged in our strengths and limitations sections under the abstract and main text that sufficient power for efficacy determination is unlikely, but this is usually not the aim of a phase I trial. Our aim is to use the secondary outcomes data and analysis for informing sample size determination in any prospective phase II trial (also mentioned in the original submission), we did

not intend to infer claims of any superiority of PAW over saline or *vice versa* in the current study. We understand that our data analysis plan might be regarded as looking to infer superiority in the original submission, which was unintentional. We have revised the data analysis plan to reflect this in the revised submission, with the following added:

"As a phase I study, the analysis of secondary efficacy indicators is likely to be underpowered due to low sample size. Therefore, the analyses of these results will not be used for statistical inference, but instead be used to perform power analysis and help determine effect sizes to inform sample size calculations for the presumed prospective phase II trial."

Specific Comments:
1. Risk of unblinding due to sensation
PAW may cause a stinging sensation or discomfort, which could lead patients or nurses to guess their group allocation. Recommendation: Record any instances of accidental unblinding and conduct a sensitivity analysis; An assessment of blinding success at the end of the trial (e.g., asking participants and the treating nurse to guess the assigned treatment).

Thank you for the advice, we agree. Your suggestion is a good approach to monitoring blinding effectiveness. We have adapted this into the revised manuscript under "Risks and benefits" with the following:

"Any instances of unblinding will be recorded, and when the participant reaches the end of the trial period, they will be asked to guess which group they were allocated to, in which they can answer (1) "control," (2) "treatment," or (3) "unsure". The blinding index will then be calculated to determine blinding effectiveness."

2. “Standard treatment” is not clearly defined
The protocol repeatedly refers to “standard care” but does not specify what it includes. The authors should provide an explicit definition, the specific components of standard treatment (such as the frequency of debridement, methods of pressure relief, and blood glucose management) should be clearly defined

Apologies for the vague description of terms such as "standard treatment" or "standard care." we have revised the definition of standard care in the "Study design" section (page 5). We would like to premise that standard care is not universal, as each wound is different and participants have various types and severities of comorbidities, requiring an individual approach to care through multidisciplinary approaches. Further, and as stated in the original submission, "There will be no restrictions to the standard care participants would usually receive. The treatment groups will not replace antibiotic treatment as an intervention for any new infections that may arise." Taking this into account, we expanded the definition of standard care in the revised manuscript and included it below:

"Standard care will involve sharp debridement as clinically needed and determined at each visit, thorough wound bed preparation and applying dressings to absorb exudate and maintain a moist wound healing environment, and weight offloading devices to relieve foot pressure if needed, following the best practice for the type, severity and stage of ulcer individualised to each participant at the clinician’s discretion, regardless of group allocation. Standard care details will be recorded."

On a related note, in the final paragraph of the introduction, we slightly altered a portion to say "PAW has never been used as a therapeutic agent for treatment of wounds" instead of "standard

care agent" as a more generalised statement and to avoid the generic "standard care" terminology as much as possible.

3. The ulcer area is too large
Inclusion requires an ulcer area between 2 cm^2 and 20 cm^2. This broad range introduces substantial heterogeneity in healing potential and infection risk. It is recommended that this be reduced, or that the baseline area be corrected in the analysis.

Thank you for raising this important methodological point. We completely agree that wound area ranging from 2 cm^2 to 20 cm^2 introduces considerable heterogeneity regarding both healing potential and infection risk.

However, because this is a phase I trial, our primary outcome is focused on evaluation the safety and tolerability of PAW. To effectively assess safety, we intentionally maintained broad inclusion criteria to ensure PAW can be safely applied without adverse reactions across a diverse and heterogeneous population of patients with diabetes-related foot wounds, including those with larger and potentially more complex ulcers.

We recognise that evaluating efficacy within this broad range is challenging. In future prospective phase II trial, where efficacy will become the primary endpoint, we intend to narrow the inclusion criteria to mitigate this variability.

In the meantime, we agree with your recommendation to correct for this variance in our current study's secondary outcomes. We have updated the data analysis plan in the revised manuscript to explicitly state, as shown below, that baseline ulcer area will be controlled for as a covariate when analysing efficacy indicators.

"One measure of efficacy will be the days to complete ulcer healing, to compare between groups by two-sample Welch's t-test if data is parametric, or Wilcoxon-Mann-Whitney test if data is non-parametric, after normalising for baseline area to correct for ulcer size heterogeneity."

References

1. Denning, D.W., *Global incidence and mortality of severe fungal disease.* Lancet Infect Dis, 2024. **24**(7): p. e428-e438. DOI: 10.1016/S1473-3099(23)00692-8.
2. Kalan, L., *et al.*, *Redefining the Chronic-Wound Microbiome: Fungal Communities Are Prevalent, Dynamic, and Associated with Delayed Healing.* mBio, 2016. **7**(5). DOI: 10.1128/mBio.01058-16.

VERSION 2 - REVIEW

Reviewer 2

Name Zhao, Yang

Affiliation China Academy of Chinese Medical Sciences Xiyuan Hospital

Date 25-Jun-2026

COI

I have reviewed the revised manuscript and your response to the reviewers' comments, and I consider that the manuscript now meets the criteria for publication.
